# The chitinolytic activity of the *Curtobacterium* sp. isolated from field-grown soybean and analysis of its genome sequence

**DOI:** 10.1371/journal.pone.0259465

**Published:** 2021-11-03

**Authors:** Ivica Dimkić, Vibha Bhardwaj, Valeria Carpentieri-Pipolo, Nemanja Kuzmanović, Giuliano Degrassi

**Affiliations:** 1 Department of Biochemistry and Molecular Biology, University of Belgrade – Faculty of Biology, Belgrade, Serbia; 2 Ras Al Khaimah Municipality Department, Director Environment Laboratories, Dubai, United Arab Emirates; 3 Embrapa Trigo, Passo Fundo, Rio Grande do Sul, Brazil; 4 Federal Research Centre for Cultivated Plants (JKI), Institute for Plant Protection in Horticulture and Forests, Julius Kühn-Institut, Braunschweig, Germany; 5 Industrial Biotechnology Group, International Centre for Genetic Engineering and Biotechnology (ICGEB), Buenos Aires, Argentina; Academia Sinica, TAIWAN

## Abstract

*Curtobacterium* sp. GD1 was isolated from leaves of conventionally grown soybean in Brazil. It was noteworthy that among all bacteria previously isolated from the same origin, only *Curtobacterium* sp. GD1 showed a strong chitinase activity. The enzyme was secreted and its production was induced by the presence of colloidal chitin in the medium. The chitinase was partially purified and characterized: molecular weight was approximately 37 kDa and specific activity 90.8 U/mg. Furthermore, *Curtobacterium* sp. GD1 genome was sequenced and analyzed. Our isolate formed a phylogenetic cluster with four other *Curtobacterium* spp. strains, with ANIb/ANIm ≥ 98%, representing a new, still non described *Curtobacterium* species. The circular genome visualization and comparison of genome sequences of strains forming new cluster indicated that most regions within their genomes were highly conserved. The gene associated with chitinase production was identified and the distribution pattern of glycosyl hydrolases genes was assessed. Also, genes associated with catabolism of structural carbohydrates such as oligosaccharides, mixed polysaccharides, plant and animal polysaccharides, as well as genes or gene clusters associated with resistance to antibiotics, toxic compounds and auxin biosynthesis subsystem products were identified. The abundance of putative glycosyl hydrolases in the genome of *Curtobacterium* sp. GD1 suggests that it has the tools for the hydrolysis of different polysaccharides. Therefore, *Curtobacterium* sp. GD1 isolated from soybean might be a bioremediator, biocontrol agent, an elicitor of the plant defense responses or simply degrader.

## Introduction

Plant diseases continue to contribute to heavy losses in the cultivation of economically important crop plants. Chemical fungicides are extensively used in current agriculture; however the excessive use has led to environmental pollution and development of pathogen resistance to fungicides. Biological control is an alternative approach to avoid the undesired effects of chemical control. Indeed, microbial antagonists that exhibit a direct action against fungal pathogens are the most widely accepted alternative approaches in plant disease management. In this context, endophytic microorganisms have attracted considerable attention for their potential biocontrol of plant diseases. Endophytic bacteria colonize the internal tissues of the plant without causing infection or negative effects on their host plant [1]. Furthermore, bacterial isolates in general can promote growth of the host plants by different mechanisms: the production of phytohormones [2], nitrogen fixation [3–5], phosphate solubilization [6] and suppression of plant diseases [7]. Therefore, they have commercially significant potential for applications as bio-inoculants, biofertilizers and biocontrol agents. Microorganisms generally express a wide variety of enzymes evaluated as a source of biocontrol agents. Among these enzymes, chitinases have received special attention due to their wide range of applications in many industrial processes and in the biocontrol of fungal plant pathogens. Chitinases are glycosyl hydrolases, able to hydrolyze 1,4 linkage of *N*-acetyl glucosamine present in chitin chains, whose size range varies between 20 kDa and about 90 kDa. There are several types of chitinases, with different specificity and biochemical characteristics. They have been classified into families based on amino acid similarity: bacterial chitinases belong to GH18 and GH19 glycosyl hydrolases families [8]. The genus *Curtobacterium* (family Microbacteriaceae) includes a wide range of bacteria isolated from different environments, such soil, cheese vat, residential carpet, and plants [9]. *Curtobacterium* spp. strains were isolated as both causative agents of plant diseases [10] and as endophytes in sugarcane [11], grapevine [12], maize [13], sorghum [14], tomato [15], coffee [16], black pepper [17], strawberry [18], citrus [19], poplar [20] and eucalyptus [21]. The genome of *Curtobacterium* sp. strain S6, recovered as endophyte from grapevine plants, was characterized for the presence of beneficial traits related to plant mineral nutrition (phosphate solubilization and siderophores), plant growth promotion (indoleacetic acid [IAA] synthesis), stress relief (1-amino-cyclopropane-1-carboxylate [ACC] deaminase and catalase activity). The presence of chitinase and phosphatase activity and the expression of a chitinase gene have been reported as mechanisms of response to disease control [9]. Although the biological significance and its possible involvement in plant defense responses against pathogens, the information regarding the distribution of chitinase genes in complete genome sequences of *Curtobacterium* spp. is still limited. In endophytic bacteria, the presence and role of chitinases have been described in a few reports. Four chitinases were characterized in the endophytic *Serratia proteamaculans* [22] and one chitinase was identified in the *Bacillus cereus* endophyte of *Sinapis* [23]. Furthermore, there is evidence about the correlation between bacterial antifungal activity and chitinase production [24].

We recently isolated autochthonous bacteria from soybean in the south of Brazil and characterized them for the presence of traits conferring rhizosphere competitiveness such as secreted enzymes (lipases, proteases and chitinases) and capacity of plant growth promotion [25]. Among all bacterial strains isolated only three showed *in vitro* chitinolytic activity when tested in laboratory in the presence of colloidal chitin: *Curtobacterium* sp. GD1, *Enterobacter cloacae* and *Staphylococcus aureus*. Considering the halo diameter of the degraded chitin around the colonies growing in a medium containing colloidal chitin, *Curtobacterium* sp. GD1 showed the highest chitinolytic activity *in vitro*. These results suggested that the chitinase was induced by chitin and secreted. Therefore, the aim of the present study is the isolation, partial purification, and biochemical characterization of the chitinase from the autochthonous *Curtobacterium* sp. strain GD1 isolated from field-grown soybean, as well as its whole genome sequencing to anchor further studies on the role of the *Curtobacterium* chitinase in the plant-bacteria interaction.

## Materials and methods

### *Curtobacterium* sp. GD1 and chitinolytic activity

The *Curtobacterium* sp. GD1 strain, isolated from surface-disinfected leaves of field grown soybean (*Glycine max* (L.) Merrill) was previously molecularly characterized by 16S rRNA gene analysis, followed by analysis of traits such as secreted enzymes (lipases, proteases and chitinases), motility, exopolysaccharides, siderophores, IAA production, antimicrobial activity, phosphate solubilization and nitrogen fixation [25]. Furthermore, the chitinolytic activity of the selected isolate was confirmed again, as described previously [25], using the *Bacillus* Minimal Medium (BMM; per liter 0.65 g of KH_2_PO_4_, 1.5 g K_2_HPO_4_, 0.25 g NaCl, 0.5 g (NH_4_)_2_SO_4_, 0.12 g MgCl_2_, 0.12 g MgSO_4_; ZnSO_4_, CaCl_2_ and FeCl_3_ were added to a final concentration of 0.01 mmol/L) with addition of colloidal chitin (1.0%) prepared from commercial chitin from shrimp shells [26]. The degradation of chitin was followed by measuring the clear halo formed around the colonies: (-) no enzymatic activity; (+) low activity, halo up to 2 mm; (++) average activity, halo from 2 to 4 mm; (+++) high activity, halo >4 mm.

### Enzyme purification and chitinase activity assay

*Curtobacterium* sp. GD1 was grown overnight in Nutrient Broth (NB) medium (per liter: 5 g peptone, 1 g beef extract, 2 g yeast extract, 5 g NaCl) at 30°C on a rotary shaker at 200 rpm. Then 500 mL of BMM with the addition of 1% colloidal chitin were inoculated with 5 ml of the overnight culture and grown for two days in the same conditions. After two days the supernatant was separated from the bacterial cells by centrifugation at 5000 × g for 15 min at 4°C. The culture supernatant was subjected to ammonium sulfate fractionation (30 and 70% saturation). The 70% pellet was resuspended in 100 mmol/L sodium phosphate (pH 7) –1.7 mol/L (NH_4_)_2_SO_4_, filtered through 0.45 μm pore-size membrane, and fractionated by hydrophobic interaction chromatography (phenyl Sepharose HP 16/10; Pharmacia Biotech), as previously described [27]. Active fractions from hydrophobic interaction chromatography were pooled and dialyzed against 20 mmol/L bis-Tris buffer, pH 7, concentrated by ultrafiltration with an YM30 membrane (Millipore) and applied to a Q Sepharose FF column, and fractionated. The chitinolytic activity after each purification step (i.e. after ammonium sulfate fractionation, as well as after the hydrophobic interaction and the ion exchange chromatography) was assayed by spotting aliquots of the suspensions onto Petri dishes containing BMM plus 0.2% colloidal chitin. The appearance of a clear halo around the spot and its diameter were the indication of the chitinase activity.

To determine the enzymatic activity of chitinase a fast-colorimetric assay based on the chromogenic substrate *p*-nitrophenyl-*β*-D-*N*,*N*’,*N*”-triacetylchitotriose [pNP-(GlcNAc)_3_] (Sigma Aldrich, USA) was used. pNP-(GlcNAc)_3_ was prepared and used as 100 mmol/L stock solutions in dimethyl sulfoxide (DMSO). The reaction mixture was composed of 200 μL of 100 mmol/L sodium phosphate buffer (pH 7.0) and 25 μL of enzyme sample. Samples were pre-incubated for 15 min at 37°C and the reaction was started by adding 2 μL of stock substrate. The reaction was terminated by adding 50 μL of 0.4 mol/L Na_2_CO_3_. Chitinase activity was determined by measuring the release of *p*-nitrophenol from the substrates pNP-(GlcNAc)_3_ reading the absorbance at 410 nm. One unit of activity was defined as the amount of enzyme required to produce 1 μmol/min product under the assay conditions.

To calculate the time of maximal activity in the cell-free culture supernatant the enzymatic activity was measured every 12 hours. The experiment was repeated three times and the standard deviation calculated. K_m_ and V_max_ were calculated for the partially purified enzyme according to the Lineweaver–Burk equation of enzyme kinetics, in the range of substrate concentrations between 3 and 90 mmol/L.

### Protein gel electrophoresis

For protein analysis and detection sodium dodecyl sulfate-polyacrylamide gel electrophoresis (SDS-PAGE) was performed on a 12% polyacrylamide gel [28]. SDS-PAGE prestained molecular weight protein markers (BioRad, USA) were used as standards. After electrophoresis proteins were visualized by staining with Coomassie Brilliant Blue R-250. Protein concentration was measured by the method of Bradford with bovine serum albumin (BSA) as a standard.

### Chitinase gene identification by Mass Spectroscopy (MS)

After SDS-PAGE analysis of chitinase-containing fractions from ion exchange chromatography, the band corresponding to the chitinase was cut out and analyzed as previously reported in Degrassi et al. [29] by using a matrix-assisted laser desorption ionization tandem time-of-flight (MALDI TOF/TOF) mass spectrometer (Model 4800; Applied Biosystem) after trypsin digestion.

### Culture conditions, DNA extraction and genome sequencing

Strain GD1 was cultured on NB medium overnight at 30°C. Bacterial cells were harvested and washed three times in 0.3% sterile NaCl. The extraction of ultra-pure DNA was done using the ZymoBIOMICS DNA Mini Kit (Zymo Research, USA), following the manufacturer protocol. The DNA yield was measured using Qubit Fluorometric Quantitation (Qubit 4 Fluorometer, Invitrogen^™^, USA). The genome of strain GD1 was sequenced using a 2 × 300 bp paired-end run (MiSeq Reagent kit v3) on a MiSeq platform, according to manufacturer’s instructions (Illumina) in commercial service (FISABIO, Valencia, Spain). Total of 1,220,080 paired reads were generated.

### Read processing, genome assembly and annotation

Reads generated by Illumina MiSeq platform were quality filtered using Cutadapt Galaxy Version 1.16.5 [30] implemented on the Galaxy Web server [31]. Quality check was performed using FastQC (http://www.bioinformatics.babraham.ac.uk/projects/fastqc/). The genome assembly of filtered reads was performed using Shovill (Galaxy Version 1.0.4+galaxy0; https://github.com/tseemann/shovill), which relies on SPAdes [32]. The genome sequences were annotated using Rapid Annotation System Technology (RAST) server [33], Prokka (Galaxy Version 1.14.5) [34] and NCBI Prokaryotic Genomes Annotation Pipeline (PGAP) [35].

### Core-genome phylogeny

*Curtobacterium* sp. GD1 sequenced in this study and 50 genome sequences of representative *Curtobacterium* spp. available in the GenBank were included into phylogenomic analysis (S1 Table). Additionally, strains *Schumannella luteola* KHIA^T^ and *Humibacter albus* DSM 18994^T^ were included as an outgroup. For phylogenomic analysis, software packages GET_HOMOLOGUES Version 11042019 [36] and GET_PHYLOMARKERS Version 2.2.8_18Nov2018 [37] were employed. Homologous gene clusters were computed from total of 53 annotated .gbk files generated by Prokka using bidirectional best-hit (BDBH), Clusters of Orthologous Groups-triangles (COGtriangles), and OrthoMCL (Markov Clustering of orthologs, OMCL) algorithms by running get_homologues.pl script implemented into GET_HOMOLOGUES software package and applying a stringent 90% coverage cut-off for BLASTP alignments (option “-C 90”). A consensus core-genome of 53 strains included into analysis was computed as the intersection of the clusters computed by the BDBH, COG-triangles and OMCL algorithms by employing script compare_clusters.pl (using option “-t 53”). The resulting core-genome clusters were processed with the GET_PHYLOMARKERS software package Version 2.2.8_18Nov2018 [37] by using a default pipeline for DNA-based phylogenies (using options “-R 1 -t DNA”).

### Whole-genome comparison

Genome sequences of the strain GD1 and related *Curtobacterium* spp. strains were compared by computing average nucleotide identity (ANI) values using the JSpecies Web Service [38]. *In silico* DNA-DNA hybridization (isDDH) values were calculated by the Genome-to-Genome Distance Calculator (GGDC 2.1; http://ggdc.dsmz.de/distcalc2.php) using the recommended BLAST+ alignment and formula 2 (identities/HSP length) [39]. BRIG (BLAST Ring Image Generator) program ver. 0.95 [40] was used for visual representation of percentage of sequence identity and sequence coverage of the genome sequence of *Curtobacterium* sp. GD1 (reference genome) and four most closely related *Curtobacterium* spp. (query sequences). The analysis was done by using the BLASTn option.

### Genome mining for GH/CBM families

Genomes of the strain GD1 and closely related *Curtobacterium* strains BH-2-1-1, MCBA15_013, MCBA15_016 and YR515 were mined for the presence of potential glycoside hydrolases (GHs) and carbohydrate binding modules (CBMs). The pangenome of these five *Curtobacterium* strains was determined using the COGtriangles and OMCL algorithms as described above, by applying Pfam domain scanning (option “-D”). A default 75% coverage cut-off for BLASTP alignments was imposed. The resulting cluster_list files were mined for the presence of Pfam IDs associated with GHs and CBMs, following the CAZy database classification scheme (http://www.cazy.org/) [8]. In order to verify the identification of Pfam families, corresponding protein sequences were additionally subjected to Pfam domain searches (database release 32.0, September 2018, 17929 entries) [41].

### *In silico* characterization of chitinase protein sequence

Conserved Domain Database (CDD, https://www.ncbi.nlm.nih.gov/Structure/cdd/cdd.shtml) [42] hits within the chitinase protein sequence were identified using CD search online web server (https://www.ncbi.nlm.nih.gov/Structure/cdd/wrpsb.cgi) [43]. Conserved amino acid residues in the active site and in the carbohydrate binding region were labeled according to GH18_PF-ChiA-like (cd06543) and Chitin-binding domain of chitinase C (cd12215) sequence clusters from the CDD. Multiple sequence alignment of sequences with cd06543 consisting of ten of the most diverse members from the cluster of sequences used to create the domain model along with the chitinase query sequence was downloaded from the CD summary page. N-terminal secretory signal sequence (N-sp) was predicted using SignalP5 server (http://www.cbs.dtu.dk/services/SignalP/) [44] using Gram positive organism group. Secondary structures were identified using the Psipred 4.0 online server (http://bioinf.cs.ucl.ac.uk/psipred/) [45]. Tertiary protein structure was estimated with AphaFold [46] by querying the protein sequence without the predicted N-sp. AphaFold was queried via UCSF ChimeraX 1.3 [47]. The generated best structure was assessed using MolProbity 4.4 [48] via SWISS-MODEL Workspace (https://swissmodel.expasy.org/assess) [49] and presented using UCSF ChimeraX 1.3 [47].

### Nucleotide sequence accession number

This Whole Genome Shotgun project has been deposited at DDBJ/ENA/GenBank under the accession JAFEVQ000000000 and BioProject number PRJNA700658. The version described in this paper is version JAFEVQ010000000. The raw sequencing reads were deposited in the Sequence Read Archive (SRA) under the same BioProject no., PRJNA700658.

## Results

*Curtobacterium* sp. GD1 previously isolated and characterized from field-grown soybean, confirmed the high chitinolytic activity *in vitro*, according to the halo diameter (> 4 mm) of the degraded chitin around the colonies growing in a medium containing colloidal chitin. This suggests that the chitinase is induced by chitin and secreted; therefore, attempts to purify the protein from the culture supernatant were carried out.

### Chitinase purification and characterization

**Fig 1:**
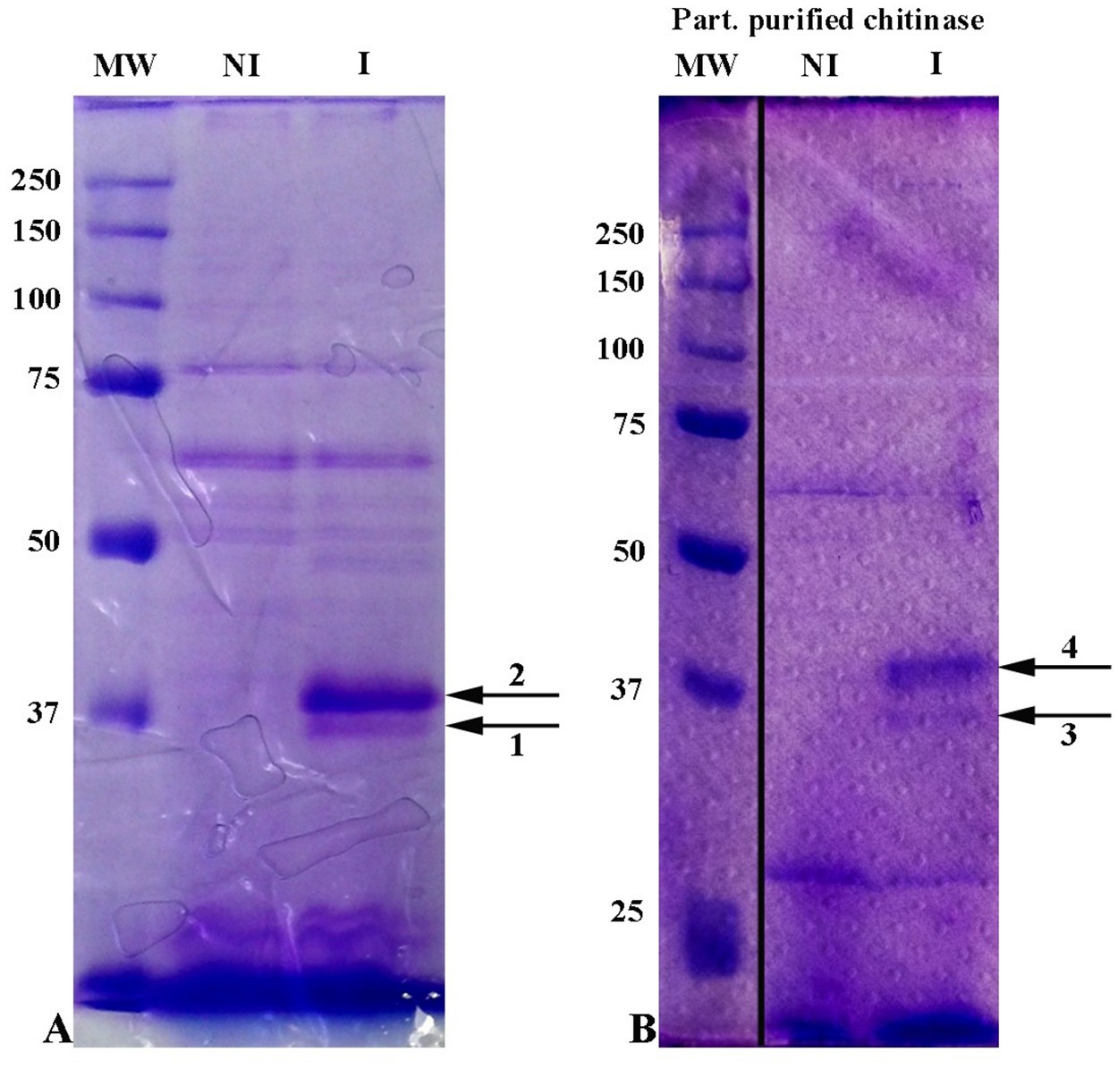
SDS PAGE analysis of supernatant of non-induced (NI) and induced (I) *Curtobacterium* cultures (A); and partially purified chitinase from ion exchange chromatography (B). Image splicing is denoted by vertical black line on the figure Fig 1B because fragments of the same original image were spliced together to remove irrelevant lanes. Fig 1A and 1B are representing different gels.

When the total proteins from the cell-free supernatants of colloidal chitin-induced and non-induced *Curtobacterium* cultures where precipitated by ammonium sulfate, the patterns of the precipitated proteins were significantly different, with two main bands present only in the induced culture, whose molecular weights were approximately 37 kDa (Fig 1A).

**Fig 2:**
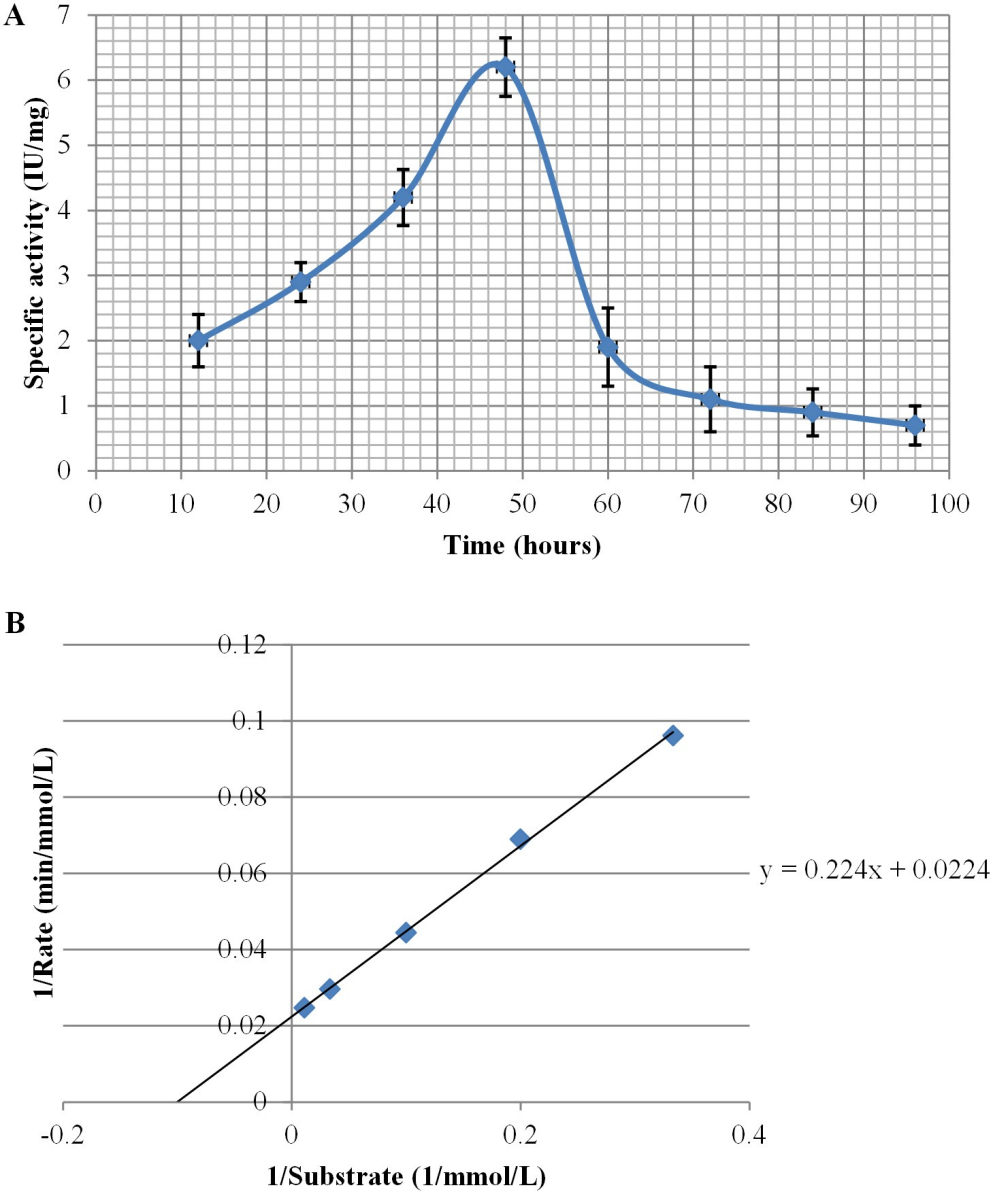
Specific activity of chitinase in *Curtobacterium* sp. GD1 cell-free culture supernatant measured at 12 hours intervals (A); and Lineweaver–Burk equation of enzyme kinetics (B).

The colloidal chitin-induced cell-free culture supernatant showed a maximum specific activity of 6.2 U/mg after 48 hours growth (Fig 2A).

The culture supernatant of a two-day colloidal chitin-induced culture was fractionated by ammonium sulfate addition. Most of the activity was found in the fractions between 30 and 70% of ammonium sulfate saturation, and the specific activity was 10.8 U/mg. Both the hydrophobic interaction and the ion exchange chromatography steps were useful to resolve the chitinase, although not to homogeneity (Fig 1B), and the specific activities were 15.4 and 90.8 U/mg, respectively (Table 1).

**Table 1:** Summary of purification of GH18 chitinase from *Curtobacterium* sp. GD1 culture supernatant.

Purification step ^a^	Total protein (mg)	Total activity (U) ^b^	Specific activity (U/mg)	Purification factor	Yield (% of activity)
Cell-free supernatant	2.77	17.20	6.20	1.00	100.00
(NH_4_)_2_SO_4_ fractionation	1.26	13.60	10.80	1.74	79.00
Phenyl Sepharose HP	0.71	10.90	15.40	2.50	63.30
Q Sepharose FF	0.08	7.26	90.80	14.60	42.20

^a^ See Material and methods for details.

^b^ Measured with *p-*nitrophenyl-β-D-N,N’,N”-triacetylchitotriose as the substrate.

K_m_ and V_max_ were calculated for the partially purified enzyme and found to be 10 mmol and 45 mmol/min, according to the Lineweaver–Burk equation of enzyme kinetics (Fig 2B). The fraction showing the peak of activity after partial purification was analyzed by SDS PAGE and contained two major bands, one of which was approximately 37 kDa (Fig 1B).

The four bands induced by chitin, indicated in Fig 1, before (bands 1 and 2, Fig 1A) and after (bands 3 and 4, Fig 1B) the purification process, were analyzed by Mass Spectrometry (MS). Bands 1 and 4 both gave peptides corresponding to a carbohydrate binding protein (CBP) that seems to be conserved in many *Curtobacterium* species indicating that *Curtobacterium* sp. GD1 has a similar catalytic activity to previously characterized enzymes of this genera. Bands 2 and 3 could be assigned to proteins of unknown function. The peptides identified by MS in the context of the full-length amino acid sequence of *Curtobacterium* sp. GD1 (accession number PZE90754.1) are: MNQNTRVR, DITVNLDWNTNVMNTAVTGTR, PGLRFSFTLATLAASDGSFGGLNSTGDATVKAIK. However, when the sequenced genome was analyzed, a carbohydrate binding protein highly similar to CBP PZE90754.1 was found in contig00004, in position 98990–100279 and showed 81% amino acid identity with PZE90754.1. Functional annotation confirmed that it is a carbohydrate-binding protein. The analysis of the annotated carbohydrate-binding protein by SignalP5 [47] pointed to the presence of a clear cleavage site between position 31 and 32, suggesting that the first 31 amino acids likely represent the signal peptide that is removed upon secretion and therefore is not present in the secreted enzyme. The theoretical and the experimental molecular weight of the secreted protein are similar; the theoretical MW without the signal peptide is 40083.25 Da, the protein band focused in the SDS PAGE slightly above the marker of 37 kDa.

### Characterization of *Curtobacterium* sp. GD1 chitinase

**Fig 3:**
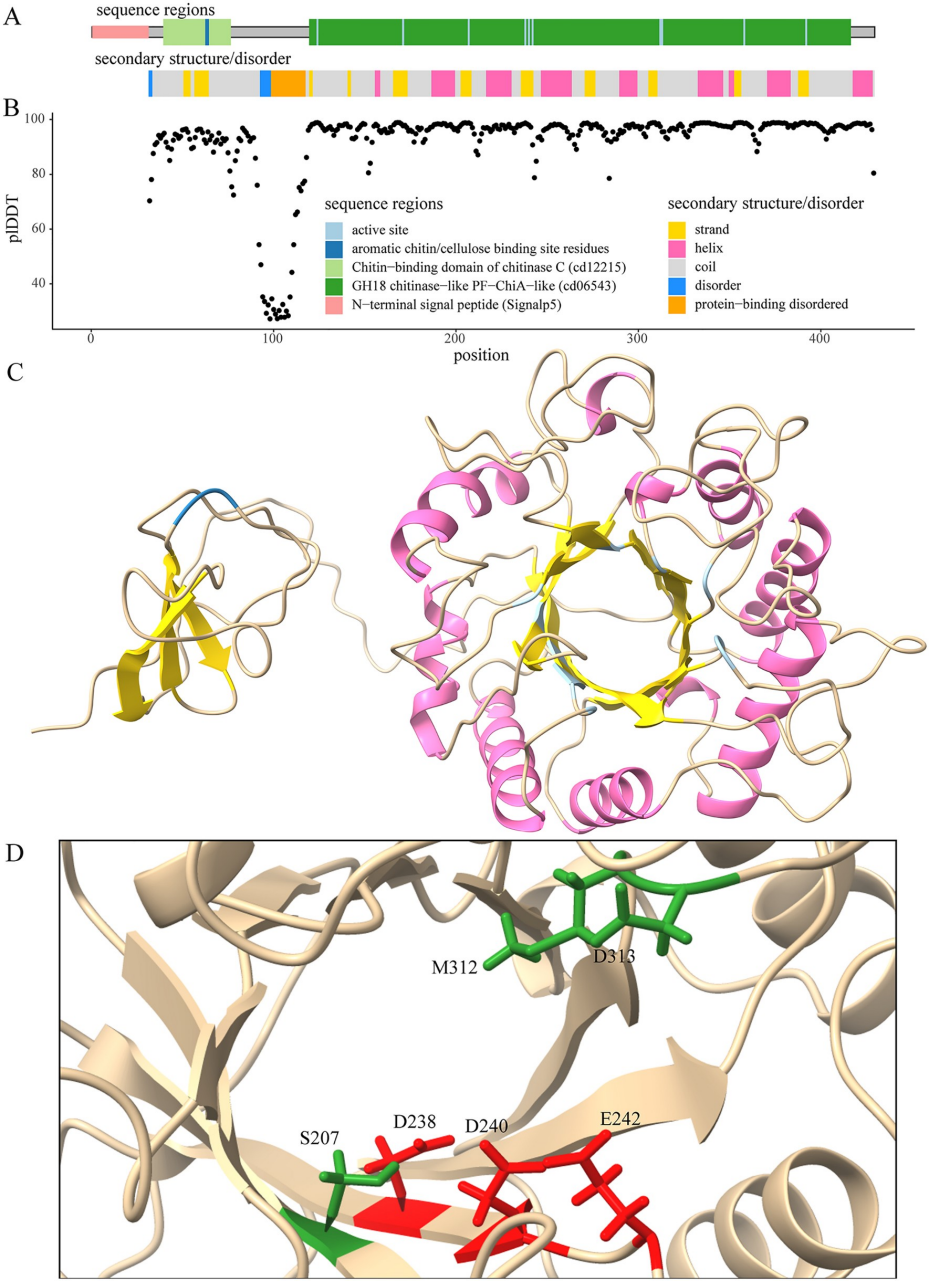
Structural characteristics of the *Curtobacterium* sp. GD1 429 amino acid chitinase protein. (**A**) Sequence region and secondary structure/disorder prediction. The protein contains a predicted 31 amino acid long N-terminal signal peptide, an N-terminal chitin binding domain (cd12215) followed by a linker and a long PF-ChiA chitinase-like domain (cd06543). In the chitin binding domain two aromatic residues (W63 and W64—numbering scheme includes the N-sp) responsible for chitin binding are shown in dark blue; In the chitinase-like domain nine regions made from ten amino acids which constitute the active site based on CDD annotation are shown in light blue (expanded figure with amino acids shown is provided as S1 Fig). The N-terminal chitin binding consists of beta strands; the linker regions is predicted to be disordered, while the chitinase-like domain consists of alternating alpha helix and beta strands connected with coiled regions. (**B**) AlphaFold per-residue confidence estimate; residues forming both domains have a relatively high confidence while the disordered linker region has low confidence. (**C**) Protein tertiary structure as predicted by AlphaFold. The aromatic amino acids in the N-terminal chitin binding domain are shown in dark blue, while the ten conserved amino acids constituting the chitinase active site are shown in light blue; helix are colored pink, while strands are colored yellow as under A. (**D**) The chitinase tunnel-like active site; amino acids residues D238, D240 and E242 (numbering scheme includes the N-sp) constituting the conserved D×D×E motif critical for activity are colored red; amino acid residues S207, M312 and D313 required for activity are colored green.

Based on CDD the *Curtobacterium* sp. GD1 chitinase contains two domains, the short N-terminal chitin binding domain (cd12215) and a long PF-ChiA chitinase-like domain (cd06543) which are connected by a linker (Fig 3A).

The protein is predicted to be excreted using Signalp5, and the N-sp is 31 amino acids long. Secondary structure and disorder prediction indicates that the N-terminal chitin binding domain consists of β-strands, while the PF-ChiA chitinase-like domain consists of eight alternating β-strands and α-helix connected with coiled regions (Fig 3A). The linker region connecting the two domains is predicted to be disordered (Fig 3A). The N-terminal chitin binding domain contains two consecutive aromatic residues (W63 and W64). The PF-ChiA chitinase-like domain contains all nine cd06543 conserved regions (consisting of 10 amino acids) which constitute the active site (S1 File). To gain insights into the possible tertiary structure of the enzyme we performed structure inference using current state of the art method AlphaFold. The best generated structure was assessed using MolProbity and is presented in Fig 3C. The predicted structure has 94.19% Ramachandran favored amino acids (S2 Fig), and the PDB file for the structure is provided in S2 File. The residue level AlphaFold confidence in the predicted conformation is mostly above 90% for the N-terminal chitin binding domain, it drops of sharply for the linker which is expected for intrinsically disordered regions, and is over 97% for the majority of the chitinase domain (Fig 3B). The predicted structure has a single β-sheet consisting of three antiparallel β-strands in the N-terminal chitin binding domain. The two solvent exposed tryptophan residues responsible for chitin binding are in the loop connecting the 2^nd^ and 3^rd^ β-strands (Fig 3C and 3D). The overall structure of the chitinase domain is a TIM-barrel (β/α)_8_-fold with a tunnel-like active site (Fig 3C). All of the ten conserved amino acid residues forming the active site according to cd06543 are located at the tunnel-like active site entrance (Fig 3C and 3D). The conserved motif D×D×E is critical (Fig 3D) and spans the 4^th^ β-strand of the TIM-barrel.

### Genome sequence of *Curtobacterium* sp. GD1

*De novo* assembly resulted in 71 contigs, with genome coverage of 140 fold and an N50 length of 115,627 bp. The total size of the draft genome sequence was 3.75 Mb, with a GC content of 71.6%, which was similar to the phylogenetically related *Curtobacterium* strains (Table 2) previously reported [50, 51]. A total of 3,601 genes were predicted by Prokka, including 3,542 coding DNA sequences, 6 rRNAs, 52 tRNAs and 1 tmRNA.

**Table 2:** Genome sequence features of *Curtobacterium* sp. GD1 and related *Curtobacterium* spp. strains*.

Strain	Source and year of isolation	Contigs (N)	N50 (Kb)	Genome size (bp)	GC content (%)	Genes**	Protein coding sequences (CDSs)**	Reference
*Curtobacterium* sp. GD1	Soybean (*Glycine max* (L.) Merrill), South Brazil, 2013	71	115,627	3,754,907	71.6	3,601	3,542	This work
*Curtobacterium* sp. BH-2-1-1	Lettuce (*Lactuca sativa* L.), Norway, 2013	CG***	CG***	3,795,948	71.4	3,677	3,616	[51]
*Curtobacterium* sp. MCBA15_013	Leaf litter, USA, 2015	219	92,686	3,948,212	71.4	3,755	3,702	[50]
*Curtobacterium* sp. MCBA15_016	Leaf litter, USA, 2015	281	63,018	3,947,873	71.5	3,675	3,622	
*Curtobacterium* sp. YR515	Not available	12	627,137	3,831,031	71.6	3,647	3,590	DOE—Joint Genome Institute, USA (Unpublished)

*Genome accession numbers are listed in S1 Table.

**Numbers based on Prokka annotations.

***Complete genome.

### Phylogenetic analysis and genome comparisons

**Fig 4:**
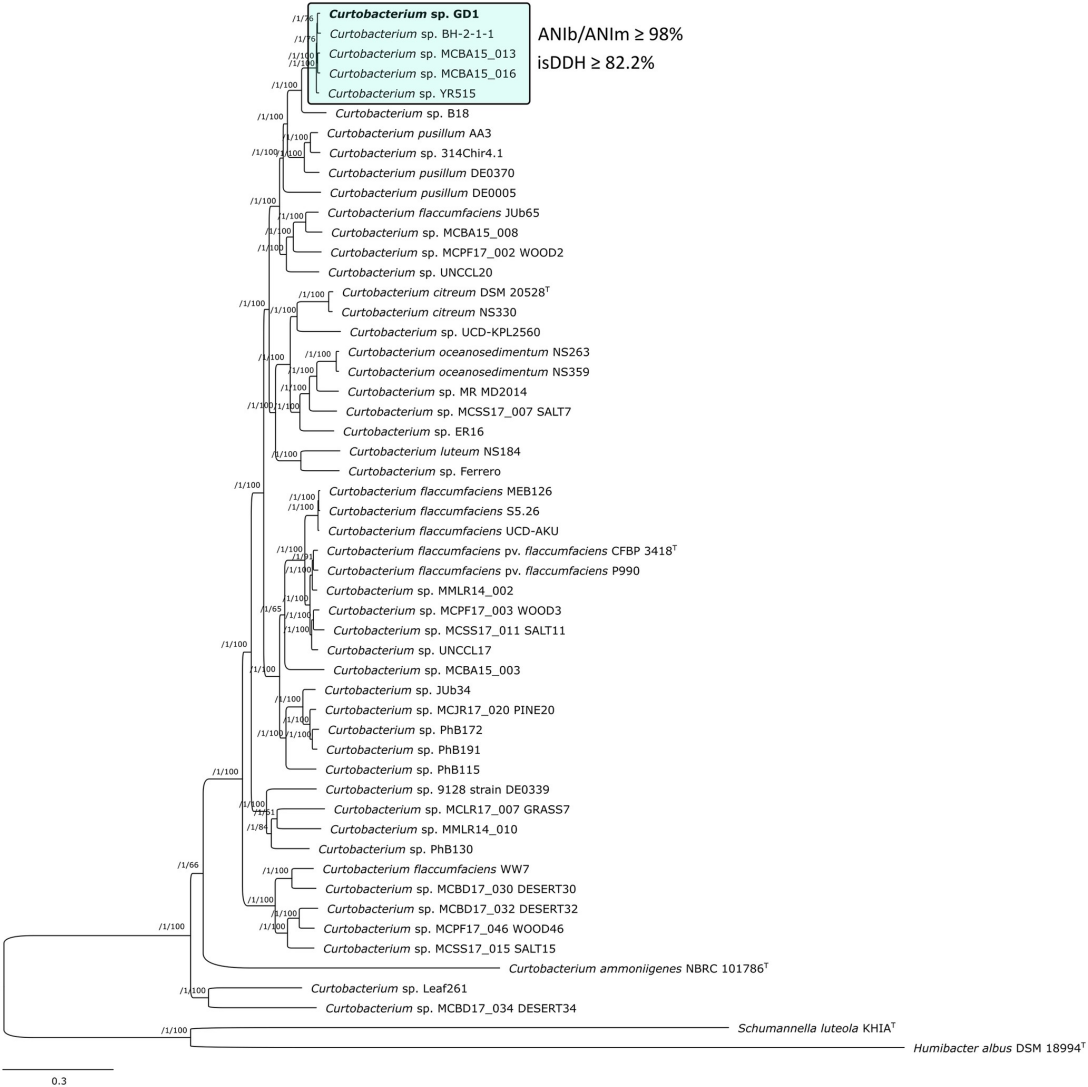
Maximum likelihood core-genome tree indicates the phylogenetic position of the strain GD1 (marked in bold) and its relationship with related *Curtobacterium* spp. The phylogenetic cluster comprising strain GD1 and representing a new and still undescribed *Curtobacterium* species is highlighted in turquoise. ANI and isDDH values calculated between members of this cluster are indicated in the figure. The tree was estimated with IQ-TREE from the concatenated alignment of 99 top-ranked genes selected using GET_PHYLOMARKERS software. The numbers on the nodes indicate the approximate Bayesian posterior probabilities support values (first value) and ultra-fast bootstrap values (second value), as implemented in IQTREE. The tree was rooted using *Schumannella luteola* KHIAT and *Humibacter albus* DSM 18994T sequences as outgroups. The scale bar represents the number of expected substitutions per site under the best-fitting GTR+F+ASC+R5 model.

A high stringency consensus core-genome contained 195 homologous gene clusters. Phylogenomic tree was inferred from 99 top markers that were selected by GET_PHYLOMARKERS software. The strain GD1 was grouped within the genus *Curtobacterium* and formed a homogenous cluster with strains BH-2-1-1, MCBA15_013, MCBA15_016 and YR515 (Table 2; Fig 4).

In order to further assess the taxonomic position of the strain GD1 within the genus *Curtobacterium*, we calculated ANI (ANIb and ANIm) and isDDH values between GD1 and closely related *Curtobacterium* spp. strains (Table 3).

**Table 3:** Average nucleotide identity (ANI) and *in silico* DNA–DNA hybridization (DDH) comparisons between GD1 and closely related *Curtobacterium* spp.

*Curtobacterium* spp.	ANI values and aligned percentages [%]		*In silico* DDH (%)
	ANIm	ANIb	
MCBA15_016	98.35 [90.04]	98.36 [85.02]	85.5
YR515	98.33 [93.68]	98.17 [89.29]	85.3
MCBA15_013	98.32 [89.49]	98.13 [84.93]	84.9
BH-2-1-1	98.17 [92.83]	98.00 [88.40]	83.6
B18	91.35 [81.24]	90.80 [75.04]	42.3

ANIm based on MUMmer ultra-rapid aligning tool; ANIb based on the BLAST algorithm.

**Fig 5:**
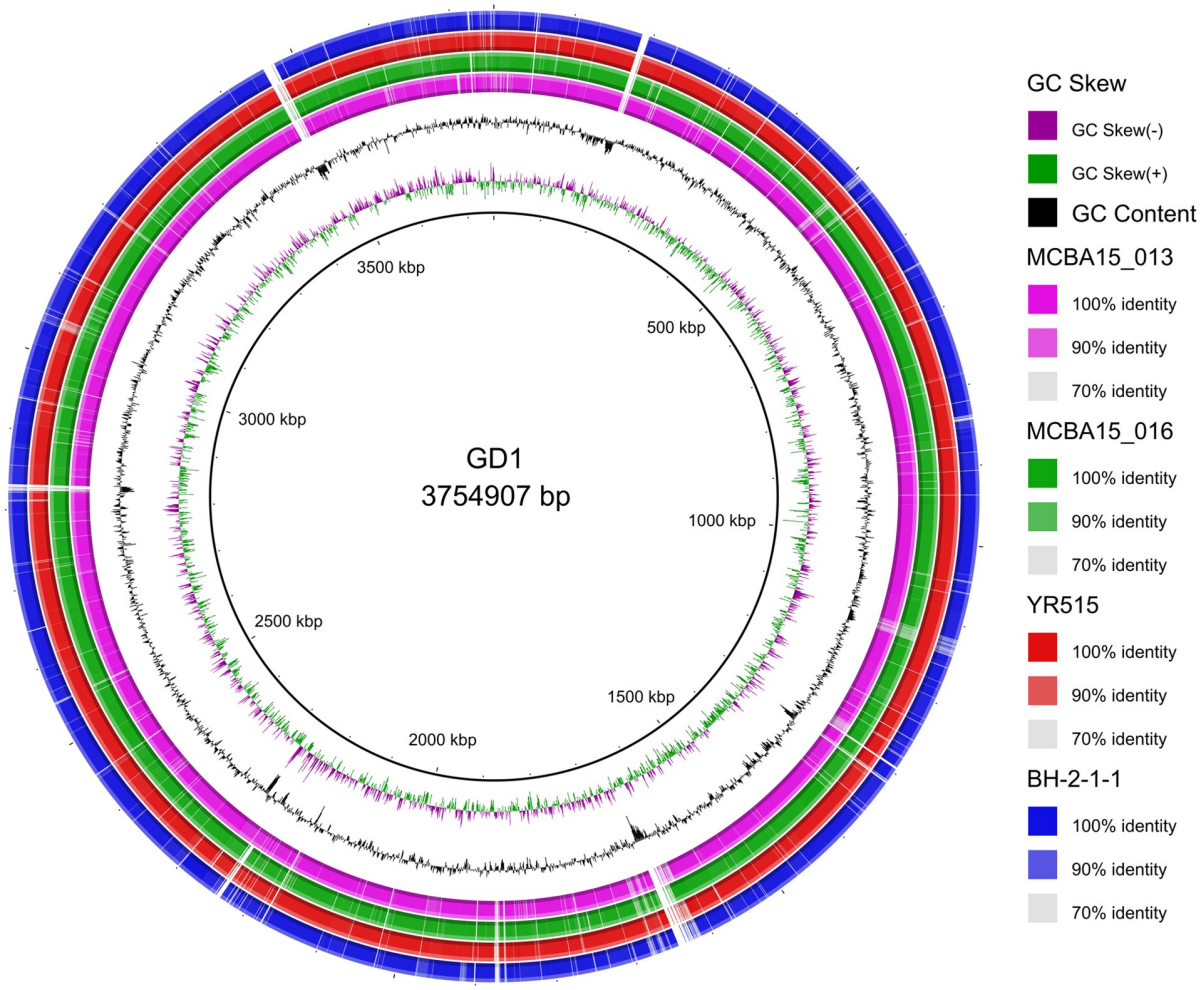
Circular representation of whole-genome sequences of *Curtobacterium* sp. GD1 and related strains from the same genus. The inner ring portrays the reference GD1 genome with corresponding genetic coordinates. The colored rings (from inner to outer ring) portray: GC skew, GC content and whole-genome sequences of strains MCBA15_013, MCBA15_016, YR515 and BH-2-1-1, as indicated in figure legend.

Values above 95–96% for ANI [52] or more than 70% for DDH [39] indicate that the strains belong to the same species. In this respect, the cluster comprising strains GD1, BH-2-1-1, MCBA15_013, MCBA15_016 and YR515 (Fig 4) represents a new, still undescribed and unnamed *Curtobacterium* species. In particular, these strains exhibited >98% ANI and >83% isDDH values (Table 3). The strain GD1 was most closely related to the strain MCBA15_016 which was isolated from the leaf litter in the USA (Table 3). Additionally, circular genome visualization and comparison of genome sequences of strains GD1, BH-2-1-1, MCBA15_013, MCBA15_016 and YR515, allowed by BRIG analysis, indicated that most regions within their genomes were highly conserved (Fig 5).

On the other hand, ANI and isDDH values suggested that strain B18 (Table 3), located on a neighboring branch (Fig 4), is distinct from GD1 suggesting that they are a separate species.

### Genomic potential for carbohydrate degradation

Furthermore, the genome sequence of GD1 was mined and compared with related *Curtobacterium* spp. strains for the presence of genes encoding carbohydrate-active enzymes (CAZymes) involved into the degradation, modification, or creation of glycosidic bonds and categorized as glycoside hydrolases (GHs) and carbohydrate-binding modules (CBMs) [53]. *Curtobacterium* strains appeared capable of targeting all substrates, in particular structural carbohydrates such as oligosaccharides, mixed polysaccharides, plant and animal polysaccharides (starch/glycogen, GH13 and CBM48 present in 11 copies), fructan, cellulose, xylan and chitin. Additionally, all genomes investigated harbored multiple copies of each protein family. Among compared strains, in almost all cases, particular GH family was present with same number of CDSs, except for the families GH16, GH23, GH29, GH35 and GH43 for which mixed polysaccharides and other plant polysaccharides were considered as a substrate (Table 4).

**Table 4:** Detected glycoside hydrolases (GH) and carbohydrate-binding modules (CBM) within related *Curtobacterium* spp. strains.

GH/CBM	PfamIDs	Main known activities	Substrate	Number of CDSs				
				GD 1	BH-2-1-1	MCBA15_013	MCBA15_016	YR515
GH1	PF00232	*β*-glucosidase (EC 3.2.1.21); *β*-galactosidase (EC 3.2.1.23); 6-phospho-*β*-galactosidase (EC 3.2.1.85); 6-phospho-*β*-glucosidase (EC 3.2.1.86); lactase-phlorizin hydrolase (EC 3.2.1.62), lactase (EC 3.2.1.108); *β*-mannosidase (EC 3.2.1.25); myrosinase (EC 3.2.1.147).	Oligosaccharides	1	1	1	1	1
GH2	PF00703, PF02836, PF02837	*β*-galactosidase (EC 3.2.1.23); *β*-mannosidase (EC 3.2.1.25); *β*-glucuronidase (EC 3.2.1.31)	Oligosaccharides	4	4	4	4	4
GH3	PF00933, PF01915	*β*-glucosidase (EC 3.2.1.21); *β*-xylosidase (EC 3.2.1.37); *N*-acetyl *β*-glucosaminidase (EC 3.2.1.52); glucan *β*-1,3-glucosidase (EC 3.2.1.58); cellodextrinase (EC 3.2.1.74); exo-1,3–1,4-glucanase (EC 3.2.1)	Oligosaccharides	2	2	2	2	2
GH4	PF02056, PF11975	6-phospho-*β*-glucosidase (EC 3.2.1.86); 6-phospho-*α*-glucosidase (EC 3.2.1.122); *α*-galactosidase (EC 3.2.1.22)	Oligosaccharides	2	2	2	2	2
GH5	PF00150, PF18564	endoglucanase (EC 3.2.1.4); *β*-mannanase (EC 3.2.1.78); exo-1,3-glucanase (EC 3.2.1.58); endo-1,6-glucanase (EC 3.2.1.75); xylanase (EC 3.2.1.8); endoglycoceramidase (EC 3.2.1.123)	Cellulose	1	1	1	1	1
GH6	PF01341	endoglucanase (EC 3.2.1.4); cellobiohydrolase (EC 3.2.1.91)	Cellulose	2	2	2	2	2
GH8	PF01270	endoglucanase (EC 3.2.1.4); lichenase (EC 3.2.1.73); chitosanase (EC 3.2.1.132)	Cellulose	1	1	1	1	1
GH13	PF00128, PF02903	*α*-amylase (EC 3.2.1.1); pullulanase (EC 3.2.1.41); cyclomaltodextrin glucanotransferase (EC 2.4.1.19); cyclomaltodextrinase (EC 3.2.1.54); trehalose-6-phosphate hydrolase (EC 3.2.1.93); oligo-*α*-glucosidase (EC 3.2.1.10); maltogenic amylase (EC 3.2.1.133); neopullulanase (EC 3.2.1.135); *α*-glucosidase (EC 3.2.1.20); maltotetraose-forming *α*-amylase (EC 3.2.1.60); isoamylase (EC 3.2.1.68); glucodextranase (EC 3.2.1.70); maltohexaose-forming *α*-amylase (EC 3.2.1.98); maltotriose-forming *α*-amylase (EC 3.2.1.116); branching enzyme (EC 2.4.1.18); trehalose synthase (EC 5.4.99.16); 4-*α*-glucanotransferase (EC 2.4.1.25); maltopentaose-forming *α*-amylase (EC 3.2.1.-); amylosucrase (EC 2.4.1.4); sucrose phosphorylase (EC 2.4.1.7); malto-oligosyltrehalose trehalohydrolase (EC 3.2.1.141); isomaltulose synthase (EC 5.4.99.11); malto-oligosyltrehalose synthase (EC 5.4.99.15); amylo-*α*-1,6-glucosidase (EC 3.2.1.33); *α*-1,4-glucan: phosphate α-maltosyltransferase (EC 2.4.99.16); amino acid transporter; [retaining] sucrose 6(F)-phosphate phosphorylase (EC 2.4.1.329); [retaining] glucosylglycerol phosphorylase (EC 2.4.1.359);; Glucosylglycerate phosphorylase (EC 2.4.1.352); [retaining] sucrose *α*-glucosidase (EC 3.2.1.48); oligosaccharide *α*-4-glucosyltransferase (EC 2.4.1.161)	Starch / Glycogen	11	11	11	11	11
GH15	PF00723	glucoamylase (EC 3.2.1.3); *α*-glucosidase (EC 3.2.1.20); glucodextranase (EC 3.2.1.70)	Starch / Glycogen	2	2	2	2	2
GH16	PF00722	lichenase; xyloglucan xyloglucosyltransferase; agarase; *κ*-carrageenase; endo-*β*-1,3-glucanase; endo-*β*-1,3–1,4-glucanase; endo-*β*-galactosidase	Other Plant Polysaccharides	5	5	4	5	5
GH18	PF00704	chitinase (EC 3.2.1.14); lysozyme (EC 3.2.1.17); endo-*β*-*N*-acetylglucosaminidase (EC 3.2.1.96); peptidoglycan hydrolase with endo-*β*-*N*-acetylglucosaminidase specificity (EC 3.2.1.-); Nod factor hydrolase (EC 3.2.1.-); xylanase inhibitor; concanavalin B; narbonin; chitodextrinase	Chitin	3	3	3	3	3
GH20	PF00728, PF02838	*β*-hexosaminidase; lacto-*N*-biosidase; *β*-1,6-*N*-acetylglucosaminidase; *β*-6-SO3-*N*-acetylglucosaminidase	Oligosaccharides	2	2	2	2	2
GH23	NA	lysozyme type G (EC 3.2.1.17); peptidoglycan lyase (EC 4.2.2.n1) also known as peptidoglycan lytic transglycosylase; chitinase (EC 3.2.1.14)	NA	4	5	4	4	4
GH26	PF02156	*β*-mannanase (EC 3.2.1.78); exo-*β*-1,4-mannobiohydrolase (EC 3.2.1.100); *β*-1,3-xylanase (EC 3.2.1.32); lichenase/endo-*β*-1,3–1,4-glucanase (EC 3.2.1.73); mannobiose-producing exo-*β-*mannanase (EC 3.2.1.-)	Other Plant Polysaccharides	1	1	1	1	1
GH29	PF01120	*α*-L-fucosidase (EC 3.2.1.51); *α*-1,3/1,4-L-fucosidase (EC 3.2.1.111)	Mixed Polysaccharides	2	2	2	1	2
GH32	PF08244, PF00251	invertase (EC 3.2.1.26); endo-inulinase (EC 3.2.1.7); *β*-2,6-fructan 6-levanbiohydrolase (EC 3.2.1.64); endo-levanase (EC 3.2.1.65); exo-inulinase (EC 3.2.1.80); fructan *β*-(2,1)-fructosidase/1-exohydrolase (EC 3.2.1.153); fructan *β*-(2,6)-fructosidase/6-exohydrolase (EC 3.2.1.154); sucrose:sucrose 1-fructosyltransferase (EC 2.4.1.99); fructan:fructan 1-fructosyltransferase (EC 2.4.1.100); sucrose:fructan 6-fructosyltransferase (EC 2.4.1.10); fructan:fructan 6G-fructosyltransferase (EC 2.4.1.243); levan fructosyltransferase (EC 2.4.1.-); [retaining] sucrose:sucrose 6-fructosyltransferase (6-SST) (EC 2.4.1.-); cycloinulo-oligosaccharide fructanotransferase (EC 2.4.1.-)	Fructan	2	2	2	2	2
GH35	PF01301	*β*-galactosidase (EC 3.2.1.23); exo-*β*-glucosaminidase (EC 3.2.1.165); exo-*β*-1,4-galactanase (EC 3.2.1.-); *β*-1,3-galactosidase (EC 3.2.1.-)	Mixed Polysaccharides	1	1	2	1	1
GH36	PF17167, PF16874, PF16875, PF02065	*α*-galactosidase (EC 3.2.1.22); *α*-*N*-acetylgalactosaminidase (EC 3.2.1.49); stachyose synthase (EC 2.4.1.67); raffinose synthase (EC 2.4.1.82)	Other Plant Polysaccharides	3	3	3	3	3
GH38	PF01074, PF07748, PF17167	*α*-mannosidase (EC 3.2.1.24); mannosyl-oligosaccharide *α*-1,2-mannosidase (EC 3.2.1.113); mannosyl-oligosaccharide *α*-1,3–1,6-mannosidase (EC 3.2.1.114); *α*-2-*O*-mannosylglycerate hydrolase (EC 3.2.1.170); mannosyl-oligosaccharide *α*-1,3-mannosidase (EC 3.2.1.-)	Other Animal Polysaccharides	2	2	2	2	2
GH42	PF02449, PF08533, PF08532	*β*-galactosidase (EC 3.2.1.23); *α*-L-arabinopyranosidase (EC 3.2.1.-)	Mixed Polysaccharides	3	3	2	3	3
GH43	PF04616	*β-*xylosidase (EC 3.2.1.37); *α*-L-arabinofuranosidase (EC 3.2.1.55); xylanase (EC 3.2.1.8); *α*-1,2-L-arabinofuranosidase (EC 3.2.1.-); exo-*α*-1,5-L-arabinofuranosidase (EC 3.2.1.-); [inverting] exo-*α*-1,5-L-arabinanase (EC 3.2.1.-); *β*-1,3-xylosidase (EC 3.2.1.-); [inverting] endo-*α*-1,5-L-arabinanase (EC 3.2.1.99); exo-*β*-1,3-galactanase (EC 3.2.1.145); *β*-D-galactofuranosidase (EC 3.2.1.146)	Other Plant Polysaccharides	1	2	2	2	2
GH51	PF06964	endoglucanase (EC 3.2.1.4); endo-*β*-1,4-xylanase (EC 3.2.1.8); *β*-xylosidase (EC 3.2.1.37); *α*-L-arabinofuranosidase (EC 3.2.1.55); lichenase/endo-*β*-1,3–1,4-glucanase (EC 3.2.1.73)	Other Plant Polysaccharides	1	1	1	1	1
GH65	PF03633	*α*,*α*-trehalase (EC 3.2.1.28); maltose phosphorylase (EC 2.4.1.8); trehalose phosphorylase (EC 2.4.1.64); kojibiose phosphorylase (EC 2.4.1.230); trehalose-6-phosphate phosphorylase (EC 2.4.1.216); nigerose phosphorylase (EC 2.4.1.279); 3-*O*-*α*-glucopyranosyl-L-rhamnose phosphorylase (EC 2.4.1.282); 2-*O*-*α*-glucopyranosylglycerol: phosphate *β*-glucosyltransferase (EC 2.4.1.-); *α*-glucosyl-1,2-*β*-galactosyl-L-hydroxylysine *α*-glucosidase (EC 3.2.1.107); 1,3-Î±-oligoglucan phosphorylase (EC 2.4.1.334)	Mixed Polysaccharides	1	1	1	1	1
GH78	PF05592	*α*-L-rhamnosidase (EC 3.2.1.40); rhamnogalacturonan *α*-L-rhamnohydrolase (EC 3.2.1.174); L-Rhap-*α*-1,3-D-Apif -specific *α*-1,3-L-rhamnosidase (EC 3.2.1.-)	Other Plant Polysaccharides	1	1	1	1	1
GH81	PF03639, PF17652	endo-*β*-1,3-glucanase (EC 3.2.1.39)	Other Plant Polysaccharides	1	1	1	1	1
GH92	PF07971, PF17678	mannosyl-oligosaccharide *α*-1,2-mannosidase (EC 3.2.1.113); mannosyl-oligosaccharide *α*-1,3-mannosidase (EC 3.2.1.-); mannosyl-oligosaccharide *α*-1,6-mannosidase (EC 3.2.1.-); *α*-mannosidase (EC 3.2.1.24); *α*-1,2-mannosidase (EC 3.2.1.-); *α*-1,3-mannosidase (EC 3.2.1.-); *α*-1,4-mannosidase (EC 3.2.1.-); mannosyl-1-phosphodiester *α*-1,P-mannosidase (EC 3.2.1.-)	Other Animal Polysaccharides	2	2	2	2	2
GH114	PF03537	endo-α-1,4-polygalactosaminidase (EC 3.2.1.109)	NA	1	1	1	1	1
GH127	PF07944	*β*-L-arabinofuranosidase (EC 3.2.1.185); 3-C-carboxy-5-deoxy-L-xylose (aceric acid) hydrolase (EC 3.2.1.-); *α*-1,3-(3,6)-anhydro-D-galactosidase (EC 3.2.1.-)	Other Plant Polysaccharides	1	1	1	1	1
GHnc	PF10129, PF06202	Likely acting as an acyltransferase enzyme	NA	2	2	2	2	2
CBM5/12	PF02839	Chitin-binding module	cChitin	1	1	1	1	1
CBM32	PF18344, PF00754	Non-reducing terminus of *N*-acetyllactosamine-binding module	NA	1	1	1	1	1
CBM48	PF02922	Gycogen-binding function	cStarch / Glycogen	5	5	5	5	5
CBM50	PF01476	Modules of approx. 50 residues found attached to various enzymes from families GH18, GH19, GH23, GH24, GH25 and GH73, i.e. enzymes cleaving either chitin or peptidoglycan.	NA	3	3	3	3	3

*highlighted rows indicated differences among the strains.

As shown in Table 4, a number of common cellulases were also detected, in particular *β*-glucosidases, *β*-galactosidase and endoglucanase (GH1-GH6, and GH8). In all compared strains (GD 1, BH-2-1-1, MCBA15_013, MCBA15_016, and YR515), presence of chitinases and chitin-binding modules were confirmed through existence of the GH18, GH23, CBM 5 and 12 families.

### Rapid Annotation System Technology (RAST) analysis

Additionally, according to the RAST server, an overview of the count of each subsystem feature and its coverage is shown on S3 Fig. The genome sequence of GD1 annotated by the RAST server shows presence of various genes or gene clusters that may be associated with resistance to antibiotics and toxic compounds. In that way it was noticed genes presence of the copper transport system and copper homeostasis together with copper chaperone with the role in copper-translocating P-type ATPase (EC 3.6.3.4), as well as copper resistance proteins CopC and CpoD, cytoplasmic copper homeostasis protein CutC and magnesium and cobalt efflux protein CorC. Also, cobalt-zinc-cadmium resistance protein CzcD and transcriptional regulator from MerR family were found as a part of a subsystem in cobalt-zinc-cadmium resistance, as well as PF00070 family (annotated in SEED database), FAD-dependent NAD(P)-disulphide oxidoreductase as a part of mercuric reductase which functions are still unclear. Interestingly, a gene involved in the uptake of selenium oxyanions (DedA protein) for later biological detoxification, was also found. Additionally, the parts of a subsystem resistance to fluoroquinolones and multi antimicrobial extrusion protein (Na(+)/drug antiporter), MATE family (energy required by efflux pumps provided by sodium ions) of MDR efflux pumps were detected. Furthermore, several genes as a part of the auxin biosynthesis subsystem product were identified in the genome of GD1.

## Discussion

*Curtobacterium* belongs to the Actinobacteria phylum and is one of those bacteria that have the potential to play a pivotal role in the decomposition and recycling of organic material [50, 54]. Although some *Curtobacterium* members were reported as soybean pathogens, such as *Curtobacterium flaccumfaciens* pv. *flaccumfaciens* as a causal agent of bacterial soybean disease [55, 56], our core-genome phylogeny evidenced that *Curtobacterium* sp. GD1 is not phylogenetically related to the type strain of *C*. *flaccumfaciens* pv. *flaccumfaciens* CFBP 3418, because they shared only 85% ANI. Furthermore, strains GD1, BH-2-1-1, MCBA15_013, YR515 and MCBA15_016 formed a cluster representing a new, still non described *Curtobacterium* species. Although other strains in this cluster mostly originating from leaf’s litter and lettuce [50, 51], this could be an indication of their omnipresence at leaves since that our strain was isolated from surface-disinfected leaves of symptomless soybean. According to the RAST server, the count of each subsystem feature and its coverage is similar as previously reported for the *Curtobacterium* sp. B2-1-1 [51]. Based on the presence of various genes or gene clusters that may be associated with resistance to the toxic compounds, GD1 has the potential to be an eco-friendly candidate for the bioremediation of toxic metal-contaminated areas as shown for other bacteria [57]. Within the genome of *Curtobacterium* sp. GD1 several genes as a part of the auxin biosynthesis subsystem were found and these genes for anthranilate phosphoribosyltransferase (EC 2.4.2.18), phosphoribosylanthranilate isomerase (EC 5.3.1.24), tryptophan synthase α and β chains (EC 4.2.1.20) were also detected in other auxins producers of microbial origin [58].

In addition, here we report the pattern of glycosyl hydrolases (GHs) from a *Curtobacterium* sp. GD1. GHs have a broad distribution among bacteria, as resulted from a comprehensive analysis of the distribution of these enzymes across all bacteria [50]. However, Actinobacteria have the highest genomic potential for being degraders of cellulose and other polysaccharides [59]. Therefore, while analyzing the genome sequence of our *Curtobacterium*, we concentrated on these GH proteins, being involved in the breakdown of large carbohydrates and playing a beneficial role in decomposition of plant residues. For instance, more efficient cellulose degradation can be achieved by means of an increase in diversity and abundance of GHs-producing microorganisms [50]. It has been reported that *Curtobacterium* spp. isolates can rapidly degrade cellulose fibers [60]. The abundance of GHs in the genome of our *Curtobacterium* sp. GD1 also suggests that it has the tools for the hydrolysis of different polysaccharides. Therefore, according to our results *Curtobacterium* seems to be a degrader. While there is large variation within the family with respect to GH richness and substrate degradation, *Curtobacterium* is one of the few genera with the potential ability to attack all identified carbohydrate substrates. In addition, *Curtobacterium* spp. isolates have the highest abundance of GHs, suggesting an increased ability to utilize and degrade a wide range of carbohydrates. This variability in carbon source usage within the *Curtobacterium* genus suggests flexibility in the ability to colonize different environments. It was reported that *Curtobacterium* may be a dominant player in the functional breakdown of dead organic material [61], playing a role as a cellulolytic bacterium [60] and that it is present in high abundance on grasses [50, 62].

The presence of chitinases in *Curtobacterium* sp. GD1 was confirmed through GH18 and GH23 families and chitin-binding modules within CBM 5 and 12 families. The enzymes belonging to these families were reported earlier to have activity on peptidoglycan, chitinases, endo-*β*-*N*-acetylglucosaminidases and some sub-families of non-hydrolytic proteins [63]. Although the presence of a chitinase gene has been reported in the genome of *Curtobacterium* isolated as an endophyte from grapevine [9] and has been suggested to be involved in plant defense responses against pathogens, leading to induced systemic resistance, there are no reports on chitinolytic activity in *Curtobacterium* spp. demonstrated *in vitro*. In this study, we report for the first time the chitinolytic activity of *Curtobacterium* sp. GD1 and show that the patterns of ammonium sulfate-precipitated proteins from colloidal chitin-induced and non-induced *Curtobacterium* cultures were significantly different, with two main bands present only in the induced culture, whose molecular weights are approximately 37 kDa, suggesting a mechanism of induction of the chitinase activity. We partially purified and biochemically characterized a chitinase from *Curtobacterium* sp. GD1 isolated from field-grown soybean. In the presence of colloidal chitin, our isolate expresses a carbohydrate binding protein, a chitinase member of glycoside hydrolase family GH18. For the two consecutive aromatic residues W63 and W64 found in the N-terminal chitin binding domain were previously shown to be responsible for chitin binding [64]. Furthermore, it is already known that TIM-barrel (β/α)_8_-fold with a tunnel-like active site is common for exo-chitinases and the conserved motif D×D×E that spans the 4^th^ β-strand of the TIM-barrel is critical for chitinase activity [65]. The activity of the D×D×E motif relies on interaction with S207, which is in some chitinases replaced by A (S1 File) [65], while M312, commonly replaced by Y in other family 18 exo-chitinase members (e.g. Tyr-214 in EQ_NAG5) [66], followed by D313 are involved in hydrogen bonding with the substrate. *Curtobacterium* sp. GD1 chitinase was able to hydrolyze colloidal chitin, as demonstrated by the large halo around the bacterial colonies and around the fractions of the protein partially purified by liquid chromatography. Therefore, *Curtobacterium* sp. GD1 secretes large amounts of a chitin-binding protein with chitinase activity, when in the presence of colloidal chitin. The possible role *in vivo* and *in planta* is still unclear and should be further investigated. However, this protein could allow *Curtobacterium* sp. GD1 to use chitin as a food source, or to be involved in antagonism and in biological control. According to previous reports, *Curtobacterium* spp. could act as a biological control agents against plant-pathogenic fungi and/or plant growth promoters such as inducers of the systemic resistance in different plant hosts, plant mineral nutrition or direct disease control agents [9, 67]. Additional investigation should be conducted to explore the potential of *Curtobacterium* chitinases in bioremediation and moreover the transformation of chitin, which is highly abundant throughout nature, into biofuel. The mechanism of regulation of the chitinase gene expression, as well as the chitinolytic activity on insoluble chitin has also to be further elucidated.

## Supporting information

S1 Fig *Curtobacterium* sp. GD1 chitinase sequence regions diagram. (TIF)

S2 Fig Ramachandran plot of AlphaFold generated structure for *Curtobacterium* sp. GD1 chitinase. (TIF)

S3 Fig Subsystem category distribution of major protein coding genes (25 most abundant subsystem categories) of *Curtobacterium* sp. strain GD1 as annotated by the RAST annotation server. The bar chart shows the subsystem coverage in percentage (blue bar corresponds to percentage of proteins included).
(TIF)

S1 Raw images Original, uncropped and minimally adjusted images supporting Fig 1. (PDF)

S1 Table List of the strains and GenBank/EMBL/DDBJ accession numbers for their nucleotide sequences used in this study. (DOCX)

S1 File Multiple sequence alignment of representative cd06543 proteins with *Curtobacterium* sp. GD1 chitinase. (HTML)

S2 File Protein data bank file of AlphaFold generated structure for *Curtobacterium* sp. GD1 chitinase. (PDB)
