# Supplementary figures and images for: The chitinolytic activity of the Curtobacterium sp. isolated from field-grown soybean and analysis of its genome sequence
Source: PLoS One. 2021 Nov 3;16(11):e0259465. doi: 10.1371/journal.pone.0259465 (PMC8565777; doi:10.1371/journal.pone.0259465)

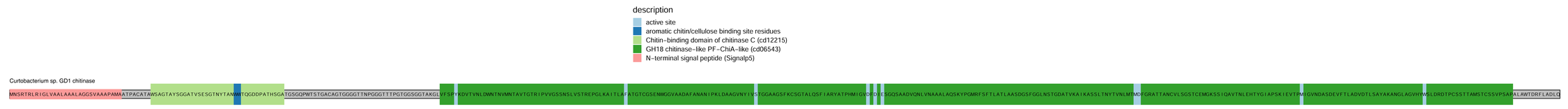

Supplement: S1 Fig — (TIF) [file pone.0259465.s001.tif]

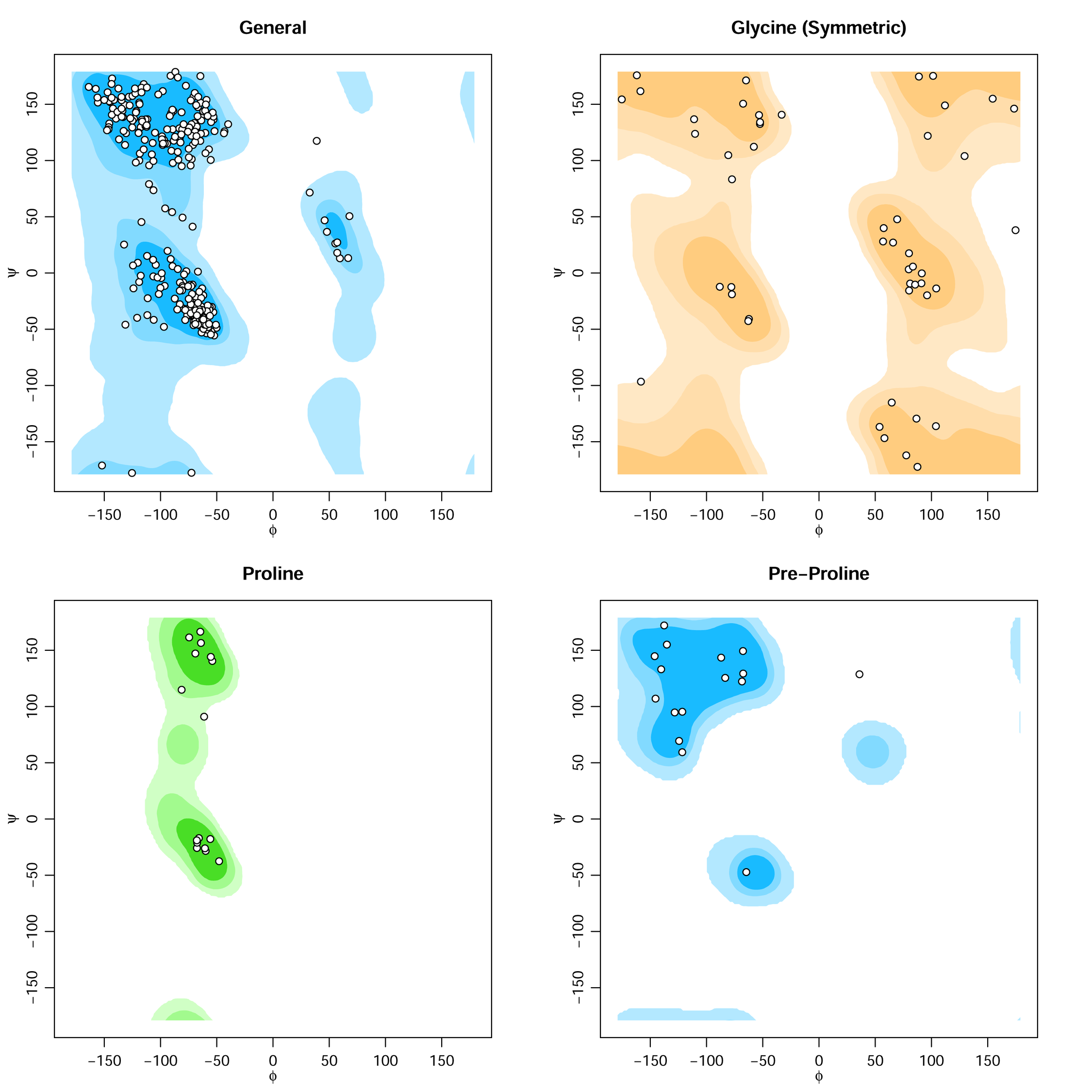

Supplement: S2 Fig — (TIF) [file pone.0259465.s002.tif]

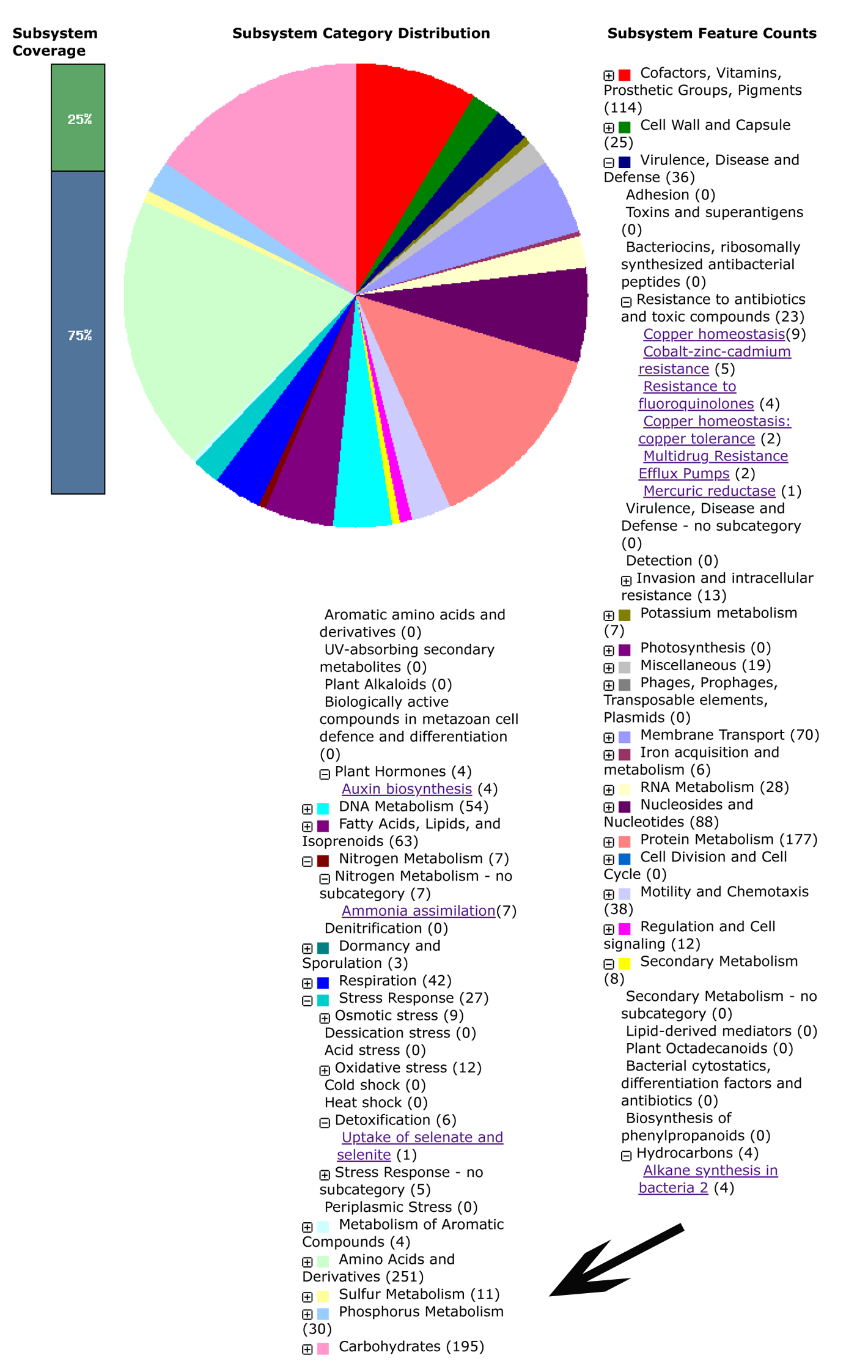

Supplement: S3 Fig — The bar chart shows the subsystem coverage in percentage (blue bar corresponds to percentage of proteins included). (TIF) [file pone.0259465.s003.tif]

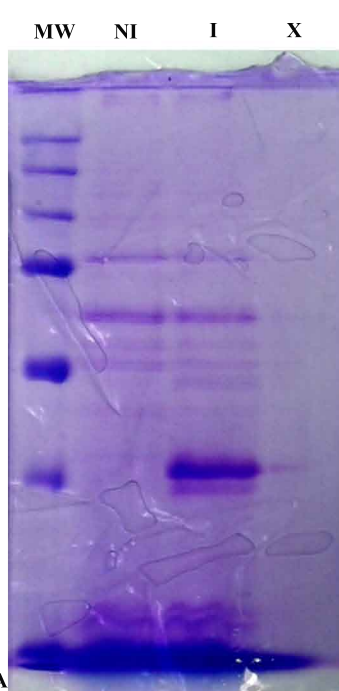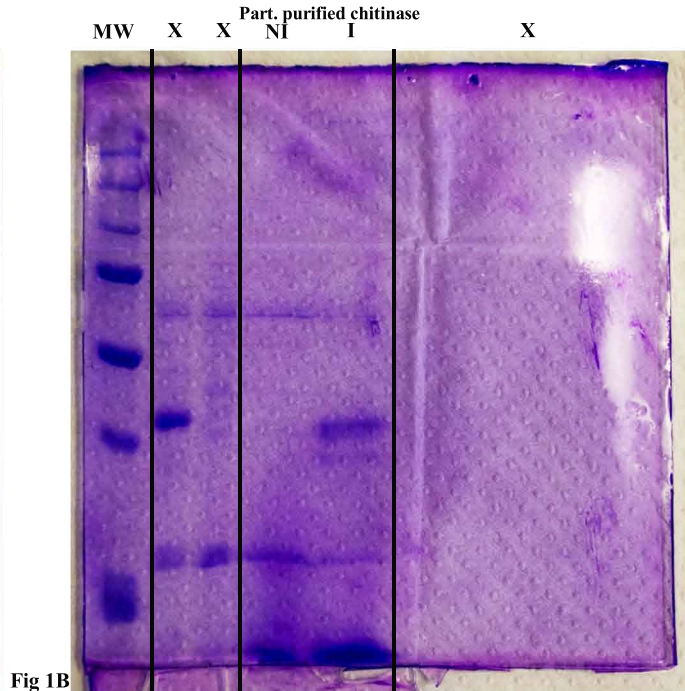

Supplement: S1 Raw images — (PDF) [file pone.0259465.s004.pdf]
