# Supplementary material for: The chitinolytic activity of the Curtobacterium sp. isolated from field-grown soybean and analysis of its genome sequence
Source: PLoS One. 2021 Nov 3;16(11):e0259465. doi: 10.1371/journal.pone.0259465 (PMC8565777; doi:10.1371/journal.pone.0259465)
Supplement: S1 Table — (DOCX) [file pone.0259465.s005.docx]

**S1 Table**

| **No.** | **Strain** | **Accession Number** |
| --- | --- | --- |
| 1 | ***Curtobacterium* sp. GD1** | JAFEVQ000000000.1 |
| 2 | *Curtobacterium ammoniigenes* NBRC 101786 | NZ_BCSV00000000.1 |
| 3 | *Curtobacterium citreum* DSM 20528 | NZ_VFMQ00000000.1 |
| 4 | *Curtobacterium citreum* NS330 | NZ_LDQB00000000.1 |
| 5 | *Curtobacterium flaccumfaciens* JUb65 | NZ_SNVW00000000.1 |
| 6 | *Curtobacterium flaccumfaciens* MEB126 | NZ_JXQU00000000.1 |
| 7 | *Curtobacterium flaccumfaciens* S5.26 | NZ_RCZL00000000.1 |
| 8 | *Curtobacterium flaccumfaciens* UCD-AKU | APJN00000000.1 |
| 9 | *Curtobacterium flaccumfaciens* WW7 | NZ_JAAOYO000000000.1 |
| 10 | *Curtobacterium flaccumfaciens* pv. *flaccumfaciens* CFBP 3418 | NZ_PUEZ00000000.1 |
| 11 | *Curtobacterium flaccumfaciens* pv. *flaccumfaciens* P990 | NZ_CP045287.2, NZ_CP045288.1-NZ_CP045290.1 |
| 12 | *Curtobacterium luteum* NS184 | NZ_LDQC00000000.1 |
| 13 | *Curtobacterium oceanosedimentum* NS263 | NZ_LDRB00000000.1 |
| 14 | *Curtobacterium oceanosedimentum* NS359 | NZ_LDRC00000000.1 |
| 15 | *Curtobacterium pusillum* AA3 | NZ_CP018783.1, NZ_CP018784.1 |
| 16 | *Curtobacterium pusillum* DE0005 | NZ_VEIY00000000.1 |
| 17 | *Curtobacterium pusillum* DE0370 | NZ_VDZH00000000.1 |
| 18 | *Curtobacterium* sp. 314Chir4.1 | NZ_OCMI00000000.1 |
| 19 | *Curtobacterium* sp. 9128 strain DE0339 | NZ_VEAF00000000.1 |
| 20 | *Curtobacterium* sp. B18 | NZ_BACZ00000000.1 |
| 21 | *Curtobacterium* sp. BH-2-1-1 | NZ_CP017580.1 |
| 22 | *Curtobacterium* sp. ER16 | NZ_MJAK00000000.1 |
| 23 | *Curtobacterium* sp. Ferrero | NZ_NXIA00000000.1 |
| 24 | *Curtobacterium* sp. JUb34 | NZ_RJVH00000000.1 |
| 25 | *Curtobacterium* sp. Leaf261 | NZ_LMMJ00000000.1 |
| 26 | *Curtobacterium* sp. MCBA15_003 | NZ_MJGJ00000000.1 |
| 27 | *Curtobacterium* sp. MCBA15_008 | NZ_MJGN00000000.1 |
| 28 | *Curtobacterium* sp. MCBA15_013 | NZ_MJGQ00000000.1 |
| 29 | *Curtobacterium* sp. MCBA15_016 | NZ_MJGR00000000.1 |
| 30 | *Curtobacterium* sp. MCBD17_030 DESERT30 | NZ_QKLI00000000.1 |
| 31 | *Curtobacterium* sp. MCBD17_032 DESERT32 | NZ_QKTO00000000.1 |
| 32 | *Curtobacterium* sp. MCBD17_034 DESERT34 | NZ_QKTN00000000.1 |
| 33 | *Curtobacterium* sp. MCJR17_020 PINE20 | NZ_QKSS00000000.1 |
| 34 | *Curtobacterium* sp. MCLR17_007 GRASS7 | NZ_QKST00000000.1 |
| 35 | *Curtobacterium* sp. MCPF17_002 WOOD2 | NZ_QKSI00000000.1 |
| 36 | *Curtobacterium* sp. MCPF17_003 WOOD3 | NZ_QKLC00000000.1 |
| 37 | *Curtobacterium* sp. MCPF17_046 WOOD46 | NZ_QKLB00000000.1 |
| 38 | *Curtobacterium* sp. MCSS17_007 SALT7 | NZ_QKSN00000000.1 |
| 39 | *Curtobacterium* sp. MCSS17_011 SALT11 | NZ_QKLE00000000.1 |
| 40 | *Curtobacterium* sp. MCSS17_015 SALT15 | NZ_QKSR00000000.1 |
| 41 | *Curtobacterium* sp. MMLR14_002 | NZ_MJGT00000000.1 |
| 42 | *Curtobacterium* sp. MMLR14_010 | NZ_MJGV00000000.1 |
| 43 | *Curtobacterium* sp. MR_MD2014 | NZ_CP009755.1 |
| 44 | *Curtobacterium* sp. PhB115 | NZ_RJKR00000000.1 |
| 45 | *Curtobacterium* sp. PhB130 | NZ_RKII00000000.1 |
| 46 | *Curtobacterium* sp. PhB172 | NZ_RKIF00000000.1 |
| 47 | *Curtobacterium* sp. PhB191 | NZ_SMBV00000000.1 |
| 48 | *Curtobacterium* sp. UCD-KPL2560 | NZ_MCIG00000000.1 |
| 49 | *Curtobacterium* sp. UNCCL17 | NZ_JMLI00000000.1 |
| 50 | *Curtobacterium* sp. UNCCL20 | NZ_FNKG00000000.1 |
| 51 | *Curtobacterium* sp. YR515 | NZ_FOOD00000000.1 |
| 52 | *Humibacter albus* DSM 18994 | NZ_ATXT00000000.1 |
| 53 | *Schumannella luteola* KHIA | VHQF00000000.1 |

**
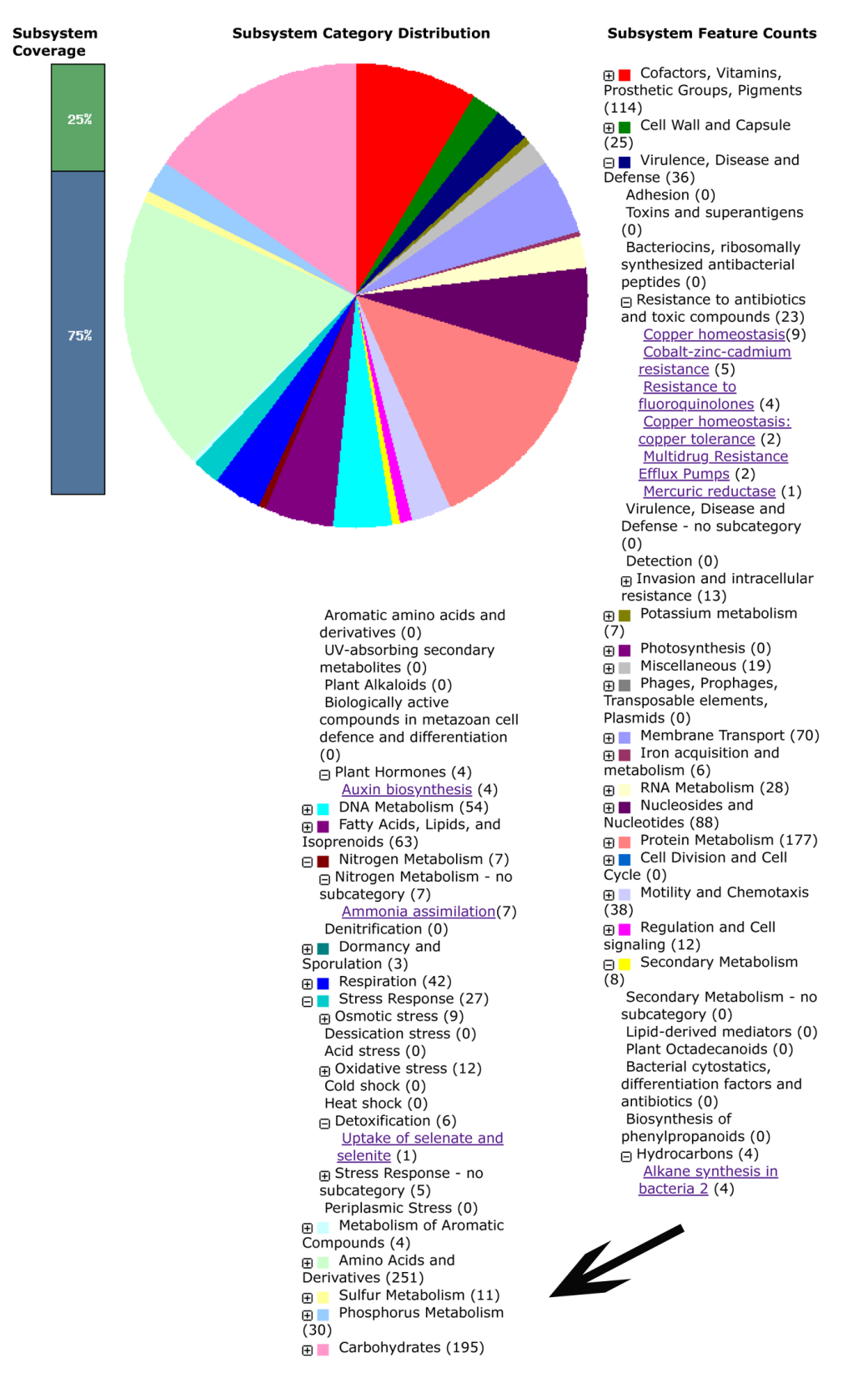
**

**Supplementary Figure 1** Subsystem category distribution of major protein coding genes (25 most abundant subsystem categories) of *Curtobacterium* sp. strain GD1 as annotated by the RAST annotation server. The bar chart shows the subsystem coverage in percentage (blue bar corresponds to percentage of proteins included).
