# Supplementary material for: The chitinolytic activity of the Curtobacterium sp. isolated from field-grown soybean and analysis of its genome sequence
Source: PLoS One. 2021 Nov 3;16(11):e0259465. doi: 10.1371/journal.pone.0259465 (PMC8565777; doi:10.1371/journal.pone.0259465)
Supplement: S1 File — (HTML) [file pone.0259465.s006.html]

```
                                                                                                   20                  40                  60                  80                 100                 120                 140                 160                 180                 200                 220                 240                 260                 280                 300                 320                 340                 360                 380                 400                 420                 440                 460                 480                 500                 520                 540                 560                 580                 600                 620                 640                 660                 680                 700                 720                 740                 760                 780                 800                 820                 840                 860                 880                 900                 920                 940                 960                 980                1000        
                                                                                 '''''''''|'''''''''|'''''''''|'''''''''|'''''''''|'''''''''|'''''''''|'''''''''|'''''''''|'''''''''|'''''''''|'''''''''|'''''''''|'''''''''|'''''''''|'''''''''|'''''''''|'''''''''|'''''''''|'''''''''|'''''''''|'''''''''|'''''''''|'''''''''|'''''''''|'''''''''|'''''''''|'''''''''|'''''''''|'''''''''|'''''''''|'''''''''|'''''''''|'''''''''|'''''''''|'''''''''|'''''''''|'''''''''|'''''''''|'''''''''|'''''''''|'''''''''|'''''''''|'''''''''|'''''''''|'''''''''|'''''''''|'''''''''|'''''''''|'''''''''|'''''''''|'''''''''|'''''''''|'''''''''|'''''''''|'''''''''|'''''''''|'''''''''|'''''''''|'''''''''|'''''''''|'''''''''|'''''''''|'''''''''|'''''''''|'''''''''|'''''''''|'''''''''|'''''''''|'''''''''|'''''''''|'''''''''|'''''''''|'''''''''|'''''''''|'''''''''|'''''''''|'''''''''|'''''''''|'''''''''|'''''''''|'''''''''|'''''''''|'''''''''|'''''''''|'''''''''|'''''''''|'''''''''|'''''''''|'''''''''|'''''''''|'''''''''|'''''''''|'''''''''|'''''''''|'''''''''|'''''''''|'''''''''|'''''''''|'''''''''|'''''''        
                                             Curtobacterium sp. GD1 chitinase    ----------------------------------------------------------------------------------------------------------------------------------------------------------------------------------------------------------------------------------------------------------------------------------------------------------------------------------------------------MNSRTRLRIGLVAALAAALAGGSVAAAPAMAATPACATAWSAGTAYSGGATVSESGTNYTANWWTQGDDPATHSGATGSGQPWTSTGACAGTGGGGTTNPGGGTTTPGTGGSGGTAKGLVFSPYKDVTVNLDWNTNVMNTAVTGTRIPVVGSSNSLVSTREPGLKAITLAFATGT---CGSENWGGVAAD---------AFANANIPKLDAAGVNYIVSTGGAAGSFK--------CSGTALQS-FIARYATPHMIGVDFDIESGQSAA--DVQNLVNAAALAQSKYP----GMRFSFTLATLAASDGSFGGLNSTGDATVKAIKASSLTNYTVNLMTMDFGRATTANCVLSGSTCEMGKSSIQAVTNLEHTYGI-------------APSKIEVTPMIGVNDASDEVFTLADVDTLSAYAKA-NGLAGVHYWSLDRDTPCSSTTAMSTCSSVPSAPALAWTDRFLADLQ-----------------------------------------------------------------------------------------------------------------------------------------------------------------------------------------------------    429    
                                                   2DSK_A|Pyrococcus furiosus    -----------------------------------------------------------------------------------------------------------------------------------------------------------------------------------------------------------------------------------------------------------------------------------------------------------------------------------------------------------------------------------------------------------------------------------------------------------------GPNANPIPEHFFAPYIDMSLSVHKPLV--------------------EYAKLTGTKYFTLAFILYSS-VYNGPAWAGSIPL---------EKFVDEVRELREIGGEVIIAFGGAVGPYLCQ----QASTPEQLAEWYIKVIDTYNATYLDFDIEAGID-----ADKLADALLIVQRERP----WVKFSFTLPSDP-----GIGLA-GGYGIIETMAKKGVRVDRVNPMTMDYYWT-----------PSNAENAIKVAENVFRQLKQIYPE----KSDEEIWKMIGLTPMIGVNDDKS-VFTLEDAQQLVDWAIQ-HKIGSLAFWSVDRDHPGPTGEVSPLHRGTNDPDWAFSHVFVKFMEAFGYTFSAQTSEASVPT-------------------------------------------------------------------------------------------------------------------------------------------------------------------------------------    311    
                                            YP_830723.1|Arthrobacter sp. FB24    ----------------------------------------------------------------------------------------------------------------------------------------------------------------------------------------------------------------------------------------------------------------------------------------------------------------------------------------------------------------------------------------------MTAINRGSDPAAKAEMEPARESVPQPVPGRFRARPQFQLSQPSSPLIAGIAVATILASVIFFWAWRSGLATGAAQATWFAGYVDVTVSPPYAFE---------------------EAATPGTRDVVLAFLVASPQDLCVASWGAAYGLDEA---NTALGLERRVSDLRSRGGSAAVSFGGARNSELAT----TCRDEGKLRTAYRDVVERYEPAFIDFDLEGADLLDQAGAERRAKAVAWLQGDRNSSGKELAVWLTLPASP------RGLTDAGIAAVEQMLQAGVDLAGVNIMTMNYGASRS-------SSQGMLDAAKAAAAATHDQLGIVYQRTGTNLSREQVWKKIGLTPMIGRNDLPGEVFELDAARDLKDYAVE-LGIQRLSMWSLNRDATCPSSEEGPTASFTCSGVDQGSFRFSDLLGSGLPGRMG----------------------------------------------------------------------------------------------------------------------------------------------------------------------------------------------    393    
                         YP_001106083.1|Saccharopolyspora erythraea NRRL 2338    -------------------------------------------------------------------------------------------------------------------------------------------------------------------------------------------------------------------------------------------------------------------------------------------------------------------------------------------------------------------------------------------------------------------------------------------MRSLPTLLAAVAAAVLLPVAAPAAGVERAATIRTAPYVDITRESPTLPE---------------------IAQATGQKHFTLAFVLGSS-AGCDPQWGGQIPLT-------EPRIVDQVTRLREMGGDVIVASGGALGPYLER----SCRTADELLAAYRKTLDAVGANHLDIDVEASIP-----HDVVNEALARLQAERG-----TEISYTLRVQSD----DTGLDPYSYQVLQSAAAHG-VDVLVNPMTMEFGSS-----------KPWGDAVIAAAESTLGQMRQIWPG----LSDAELKARLGVTPMIGRNFNGK-VFDQSHARQLVDWAAA-NRIGLLSFWSAGRDNGRCPGGPVAPDCSSIAQSDYEFTRIFGAFAG-----------------------------------------------------------------------------------------------------------------------------------------------------------------------------------------------------    318    
                                YP_001415787.1|Xanthobacter autotrophicus Py2    MATRTITWAWGAQEVVSFDPATDVLDFGWLNASDFTITEVNGSVVIALPANAHSYTLEGVSLSDLSLANISAFDASVFAQWSAAIAADGATTPVAGTLFALSAARDTDTVLSFNPAADKLDFSGLSASDFSIAEVNGSVVIYLSAERQTYILDGVTLSQLSLSSIASQDAAVLAEWSQALASPAGSSAAPTSGGGEAVTTTLGWNWNTQTVLAFDPARDKIDFGWLTPQDFSVVELNGSVVILMPGAQQSYTLEGVTLAELSAANITARSAVVLEEWATLLSGASGSGGGSGGGSGSTLPVIEASAWIDAKAYVAGDLVSVGHLVYQANWWTLGTDPTSDHGAVGTGHVWSVVGYADLTPVAPDAPDGLHLLTTTETSATLTWDAAEVSGVGTVTGYAIYRDGELVGTTTDLSFKVSGLVADTTYHFSVVAVDEAGTSPAATPIAVTTDAPGTDAADQQHFSPYFEMWLPSSQNLV--------------------QTVEDVGLTAVTLAFVLGT--GPDQIGWGGLGSIDNDT-LANGTTISSMVAALQQNGVEVTISFGGGYGQEPAL----SFTNVAQLTAAYQSVMDKYNVTSLDFDIEADALTNTAASHLRNEALVALEQANP----DLTVSFTLPALP------TGLNQAGLDLLAQAKADGVEIDTVNIMVMNYGAYY--------DSGDMGKDAIDAAEATIAQLHQL-----------GLDAKVAITPMIGQNDVPGEVFTLDDARQLLDYAEGNDHIAYIAMWSLGRDHGDDVGHLTETSSGVAQHDYDFAKIFSMV--------------------------------------------------------------------------------------------------------------------------------------------------------------------------------------------------------    751    
                                ZP_02960160.2|Providencia stuartii ATCC 25827    -----------------------------------------------------------------------------------------------------------------------------------------------------------------------------------------------------------------------------------------------------------------------------------------------------------------------------------------------------------------------------------------------------------------------------------------------------------MAKIPPIQGEMMMNKTLFAPYLDVTVDAIWNDPLSPNGK--------PNPKYSQAAISHSVDGVYLAFLTAD--PNNNAAWGAYSSMP-------IAWAKPFCDALIEANIKVIVSFGGAANYDVS-----ARQTVDQLIATYQEVIDILGASQLDFDFENDLYD----ADKTFTALSTIVKNNP----DITLSLTLPVMP------YGLVEKGLALVQKSVNAG-LKMKVNGMAMDYGQGT---------DKNMGQAAVDVATSLKNQLKTYYPT----LTDAELYDLVQVTPMIGLNDDRS-MFNFNDINTLSNFAKT-NGVNLISMWSLTRDRVGVGESASASYSGNPEQTKDFEYTERFTNALK----------------------------------------------------------------------------------------------------------------------------------------------------------------------------------------------------    316    
                             NP_901110.1|Chromobacterium violaceum ATCC 12472    -------------------------------------------------------------------------------------------------------------------------------------------------------------------------------------------------------------------------------------------------------------------------------------------------------------------------------------------------------------------------------------------------------------------------------------------------MMKRFAALSALAAAAAGLASPAAQAGAFAPYVDMTLWPTPQID--------------------KLGVNQGIQQFTLAFVVAK--GGCAPSWGGVLAIPGSGSDQQLSAIRNGINNFRGKGGEVMVSFGGANGTPLQQ----ACTTNASLQAAYQTVLDTYNLSRIDFDIEGGAQTDTAANNRNFAVVAALQKNYKAKGKTLHVSLTLPAMP------FGLTQDGQRVLASALANGVALDTVNIMAMDYGQS----------NPNMGAAAKQAAQALYSQIDAAYKAHGQTLTDAQLWQKVGVTPMVGLNDTQPETFTVDNAKDLYGMANS-NRFGLLSMWSISRDKSCPNNGHYVDAQCSGIVQTPYAFSNVFKGFKDHWGSGVTQDPNYGGGDNGNGGGPVNGQPWSSGQVYNTGNTVTYSGATWKAQWWTQGDVPGQASVWQQQGGGLQQWSATAAYSAKDCALYQGKKYCAKWWTQGNLPAAGDPWVLSN---------------------------------------------------------------------------------    450    
                                    EAY61932.1|Burkholderia cenocepacia PC184    ---------------------------------------------------------------------------------------------------------------------------------------------------------------------------------------------------------------------------------------------------------------------------------------------------------------------------------------------------------------------------------------------------------------------------------------------------------------------MSHGAGAYAPYVDVTLYPTPLVD--------------------QIGVRQGIQQFTLAFVVAG--NGCVPSWGGVQPIGNGASGGLLTALSTSIASYRAKGGEVAVSFGGANGTPLMQ----ACSTVPALKSTYQTVIDTYGLTHIDFDIEGASQQDTAAVARNFQAVAQLQADYAAKGKPLHVTLTLPTMP------TGLTQDGVNVVNAAIANKTTFDAVNVMAMDYGPA----------NIDMGAAAISAAQALYSQLDTAFKSAGQPKTNAQLWQMVGVTPMIGVNDVQGETFTLANAQTVLNAAIA-NGYGFFGNWSVGRDQACPSGGTYASPTCSGVAQQPYAFAAIFKKLDGKWGAGVTQDPNYGGGSDGGTPQPGAPWAAGQVYTAGATVTYQGTTYQAQWWTQGDVPGQAAVWKPIGGGTPAWSATTAYQGGTCVTRGGPFPAWQDRTPGRPAADAGARRCPTRARARPAAVPSRRRAAPRACRDAGESARSARRPTARTSRRRASAPPRAPTARARSRRSSRPSRVRETARSARRAPRGRPPSHR    511    
    YP_096229.1|Legionella pneumophila subsp. pneumophila str. Philadelphia 1    ------------------------------------------------------------------------------------------------------------------------------------------------------------------------------------------------------------------------------------------------------------------------------------------------------------------------------------------------------------------------------------------------------------------------------------------------MRYRRFLCLLNGMLLSSNVAAFSPSAPIFSPYVDLTINTHWDSQSQDME----------PMDLISPAKKLGIKAYHLAFITDS--GQCQPAWGAQQNYSV-----AKGWGKKQFDTLSREGVKLTVSFGGASGTDIS-----YHCDKNQLINTFSQVANQYHANVLDFDIENGTAN----IPNLLQALKLFQKEHP----DVLLSFTLPVMP------EGLTSVGKEIITSAATLG-LHFNVNIMAMDYGPAY---------SGDMGDYAISAATNLHQFLQEIYPD----KKPEALWQMIEVTPMIGVNDVNTEQFTLSNAAQLKQFAQK-NLLGGLSMWSFNRDKPCADKWASPVCSGNNLQSHDYEFVKYFQ--------------------------------------------------------------------------------------------------------------------------------------------------------------------------------------------------------    324    
                                       YP_001850315.1|Mycobacterium marinum M    ----------------------------------------------------------------------------------------------------------------------MPSYVFATPEALTTVSSDLAGIGIAIRSANLTAAPSTTQVLAAAQDEVSAAIAGFFSGHAQQFQTLSAQASAFHDQFVETLSGASGAYAAAEAASTSPLQNLEQSLLAVINAPSQALTGRPLIGDGANGSPGTGQNGGDGGWLWGNGGNGGSGAPGGAGGAGGSAGLWGRGGDGGVGGDATIAGGPGGNGGAGGANGLIGGGNGGAGGAGGAGAPGGDIAGGTGGAGGIGGANRQLLSLDGTGGAGGTGGGGGFGGIGAAGGDAGAGGAGGANQALLGGTGGTGGNGGNGGAGGAGGGLGGQGGVGGTGGVNHALLGGTGGHNGLNGSNGSDGITGTGSTGVYKPYVDITLWPYPDGSGY----------------NFSDAANAGITDVTLAFITADT-TNGQAAWGGYTAYDVTG-GSQISYIENQITNMTNAGINGTISFGGQAGTPLAVYAANNSLTAAQLAAQYQEVMSTYGIYSIDFDDEGAILTNSSALTLQAQAIALSQAWGTANGTPVTVSYTVPVAP------SGLTAEGMAPINAAISSGVNVSTVNIMAMDYYDG----------TTQMGTAAIDAATATHGQLMTLYPS----LSSDQAWAMLGVTPMIGVNDDTSEIFTLTDAQTLTSFAQD-NNIGQLSMWQLPRDQTGDIGVSNNNGSGVEQTPFEFSEIFEQYASNS----------------------------------------------------------------------------------------------------------------------------------------------------------------------------------------------------    654    
                                                                                         
                                                                    Consensus    MATRTITWAWGAQEVVSFDPATDVLDFGWLNASDFTITEVNGSVVIALPANAHSYTLEGVSLSDLSLANISAFDASVFAQWSAAIAADGATTPVAGTLFALSAARDTDTVLSFNPAADXXXXXXXXXXXXXXXXXBXXXXXXXXXXXXXXXXXXXTXXXXXXXXXXSXXXAXXXXXXXXXXXXXXXXAXXXXXXXXXXXXXXXXXXXXXXXXXXXPXXBXXXXXXXXXXXXSXXXXXXXXXXXXXXXXXXXXXXGXXXXXLXXXXXXXXSXXXXXXXXXXXSXXXXXXGGXGGXXGXXXXXXXXXXXXXAXXXXXXXXXXXGXXXXXXXXXXXGXDXXXXXXXXXXXXXXXXXXXXXXXXXXXXXXXXXXXXXXXXXXXXXXXXXXXXXXXXXXXXXXXXXXXXXXXXXXXXXXXXXXXXXXXXXXXXXXXXXXXXXXXXXXXXXXXXXXXXXXXXXXXXXXXYXXXXXXXXXXXX+++++++++++++++++++++XXXXXXXXXXXLAFXXXX+++XXXXXWXXXXXX+++++++++XXXXXXXXXXXXXXXXXXXXXGGXXXXXX++++++++XXXXXLXX+XXXXXXXXXXXXXDXDXEXXXX+++++XXXXXXXXXXXXXXXX+++++XXXXXTXXXXX++++++XGLX+XXXXXXXXXXXXX+XXXXVNXMXMXXXXX+++++++++++XXXXXXXXXXXXXXXXXXXX+++++++++++++XXXXXXXTPMXGXNXXXX+XFXXXXXXXXXXXAXX+XXXXXXXXWXXXRDXXXXXXXXXXXXXXXXXXXXXXXXXXXXXXXXXXXXXXXXXXXXXXXXXBGXXXXPXXXXXXXXXXXXXXXXTXXXXXXXXXXXXXXXXXXXXXXXXXXXXGXXXXXXXXXXXXXXXXXXXXXXXXXXXXXXXXXXXXAXXXXXXXXXRARPAAVPSRRRAAPRACRDAGESARSARRPTARTSRRRASAPPRAPTARARSRRSSRPSRVRETARSARRAPRGRPPSHR    1007
```
